# Developing Enzyme Immobilization with Fibrous Membranes: Longevity and Characterization Considerations

**DOI:** 10.3390/membranes13050532

**Published:** 2023-05-20

**Authors:** Yue Yuan, Jialong Shen, Sonja Salmon

**Affiliations:** 1 Center for Nanophase Materials and Sciences, Oak Ridge National Laboratory, Oak Ridge, TN 37831, USA; yuany@ornl.gov; 2 Fiber and Polymer Science Program, Department of Textile Engineering Chemistry & Science, North Carolina State University, Raleigh, NC 27695, USA; jshen3@ncsu.edu

**Keywords:** enzyme, immobilization, biocatalyst, longevity, performance

## Abstract

Fibrous membranes offer broad opportunities to deploy immobilized enzymes in new reactor and application designs, including multiphase continuous flow-through reactions. Enzyme immobilization is a technology strategy that simplifies the separation of otherwise soluble catalytic proteins from liquid reaction media and imparts stabilization and performance enhancement. Flexible immobilization matrices made from fibers have versatile physical attributes, such as high surface area, light weight, and controllable porosity, which give them membrane-like characteristics, while simultaneously providing good mechanical properties for creating functional filters, sensors, scaffolds, and other interface-active biocatalytic materials. This review examines immobilization strategies for enzymes on fibrous membrane-like polymeric supports involving all three fundamental mechanisms of post-immobilization, incorporation, and coating. Post-immobilization offers an infinite selection of matrix materials, but may encounter loading and durability issues, while incorporation offers longevity but has more limited material options and may present mass transfer obstacles. Coating techniques on fibrous materials at different geometric scales are a growing trend in making membranes that integrate biocatalytic functionality with versatile physical supports. Biocatalytic performance parameters and characterization techniques for immobilized enzymes are described, including several emerging techniques of special relevance for fibrous immobilized enzymes. Diverse application examples from the literature, focusing on fibrous matrices, are summarized, and biocatalyst longevity is emphasized as a critical performance parameter that needs increased attention to advance concepts from lab scale to broader utilization. This consolidation of fabrication, performance measurement, and characterization techniques, with guiding examples highlighted, is intended to inspire future innovations in enzyme immobilization with fibrous membranes and expand their uses in novel reactors and processes.

## 1. Introduction

**Figure 1:**
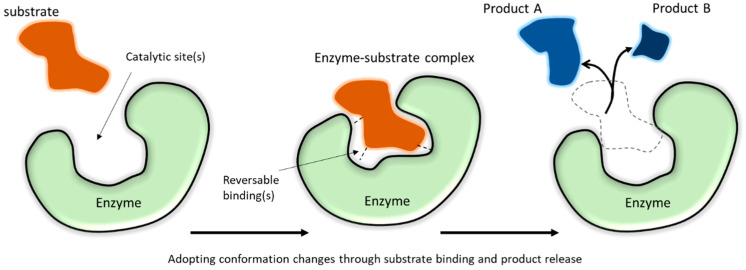
An illustration of enzyme catalytic function.

Enzymes are protein-based catalysts that are present in all viable biological systems. In nature, enzymes are soluble or membrane-bound, depending on their role. Their catalytic functions depend on active site chemistry, molecular structure, and conformation (Figure 1). As true catalysts, enzymes participate in but are not consumed by the reaction. Due to the highly selective reactions catalyzed by different enzyme classes (Table 1), enzymes are useful for a broad range of consumer, medical, and industrial applications. Many commercial enzyme-catalyzed reactions are carried out in liquid environments, as batch reactions, using soluble (or “free”) enzymes mixed with the reaction liquid. While this approach is simple, large amounts of enzyme may be needed, enzymes are disposed when the process is complete, and enzymes may remain in the product. Alternatively, enzymes can be attached (or “immobilized”) to insoluble supports or formed as insoluble complexes. Immobilization allows enzymes to be recycled, allows enzymes to be fixed in a particular reaction zone to achieve a specific type of performance, and allows enzymes to be easily filtered or separated from reaction products, which can enhance product quality. Immobilization can improve enzyme stability and create useful physical forms for controllable processing [1,2,3,4,5,6,7], for use in sensors [8,9,10,11] and biomedical devices or accessories [12,13,14], and for selective filtration or separation materials [13,15,16]. Enzyme immobilization has also been used to make model systems for exploring protein-material interactions [17,18] and to better understand biological systems, with the potential to copy features of these systems by mimicking the cellular environment [19].

**Figure 2:**
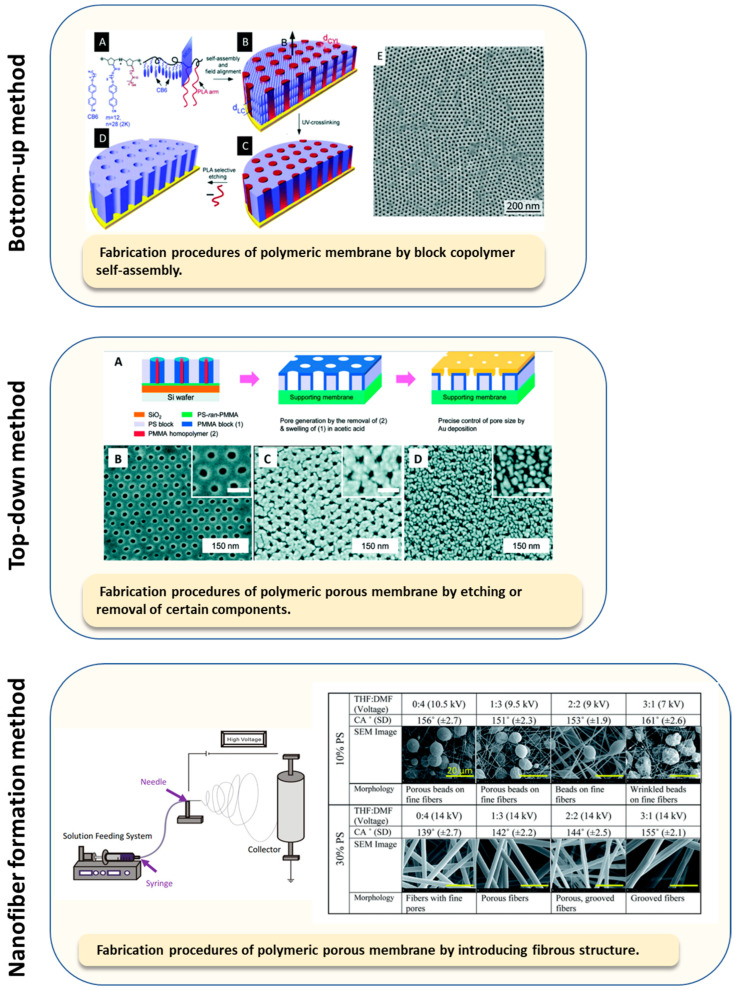
Examples of porous polymer membrane fabrication techniques [23], showing porosity created by bottom-up methods, such as polymer assembly (adapted with permission from [24], Copyright 2010, American Chemical Society, and from [25], Copyright 2014 John Wiley and Sons.), by top-down methods such as etching or dissolving certain components (adapted with permission from [26], Copyright 2010, American Chemical Society), and by assembling fine fiber structures, such as by electrospinning (adapted from [27], which has an open-access CC BY-NC 3.0 license).

Combining enzymes with membranes as the immobilization support can augment enzyme performance in synergy with the fundamental selective permeability function of the membrane. Membrane permeability is controlled by porosity, which can be created in various ways using polymeric materials (Figure 2). Techniques include phase inversion, in which a polymer is induced to precipitate from an initially homogeneous film by evaporation, cooling, or exposure to a nonsolvent, and electrospinning, where polymers are extruded into nanofibers on a collector to create interconnected open pores, ranging in size from several micrometers down to tens of nanometers [21]. Composite and nanocomposite membranes, made by dip-coating, grafting, phase inversion, and other methods, enhance the performance of conventional membranes by incorporating supporting layers or components that enhance the mechanical strength, selectivity, permeability, and stability [21]. The optimal pore size depends on the application requirements. Whereas dense membranes, such as those used for pervaporation, dialysis, and osmosis, are considered nonporous (pore size < 1 nm), porous membranes are used for microfiltration (MF, pore size 0.1–10 μm), ultrafiltration (UF, pore size 10–100 nm), and membrane distillation (MD, pore size 0.2–1 μm) [21]. Notably, the pore size range in UF membranes matches the pore size range identified as generally favorable for achieving high enzyme loading during immobilization [22].

Materials with a high surface area, such as membranes, can augment the enzyme performance by reducing mass transfer barriers, while the selective reactivity of enzymes can enhance membrane functionality by transforming substrate molecules into chemical forms that preferentially permeate the membrane. To complement prior reviews [1,2,28,29,30] that focused on specific enzymes [29,31] or specific materials [28,32], this review emphasizes synergies between enzymes, as efficient reaction catalysts, and fibrous membranes, that contribute versatile physical separation and durable physical support functionality, including numerous fabrication options, dimensions, geometries, and chemo-physical properties. Methods used to characterize these materials and evaluate their catalytic performance are summarized, along with examples that are intended to inspire new solutions to complex technical challenges in healthcare, energy and fuel production, waste management, and climate change mitigation.

## 2. Functional Attributes of Enzymes

Enzymes are compact globular proteins with catalytic active sites that lower the transition state energy for specific chemical reactions to occur [33], that in some cases would take hundreds or thousands of years to occur if not catalyzed [34]. Enzymes range in physical size from small monomers (with single protein chain domains), such as hen egg white lysozyme that has a molecular weight of 14.3 kDa [35] and a spherical shape with a diameter of around 3 nm [36], to large multimers (having multiple protein chain domains that form a complex), such as tetrameric beef liver catalase that has a molecular weight of ~232 kDa [37] and a diameter of at least 8 nm [38,39]. As is characteristic of proteins in general, enzymes exhibit self-assembly phenomena, have a density near 1.37 g cm^−1^ [38], tend to have more charged amino acid residues at their surface than in their interior [40], and, when solvated, are intimately surrounded by 3 to 4 layers of hydrating water (with a thickness around 0.7–1 nm) [41]. Often, but not always, the substrate size is small compared to the enzyme molecule, allowing substrates to diffuse into the enzyme active site. For example, bovine α-carbonic anhydrase has a molecular weight of ~30 kDa, while the substrate CO_2_ is about 44 Da, with a weight ratio of about 680:1 [42], and catalase is more than 6000 times larger than its substrate, hydrogen peroxide (34 Da) [43]. The catalytic active sites in enzymes are formed by precise spatial relationships of chemically reactive amino acid side chains through correct folding of the protein polymer [33].

**Figure 3:**
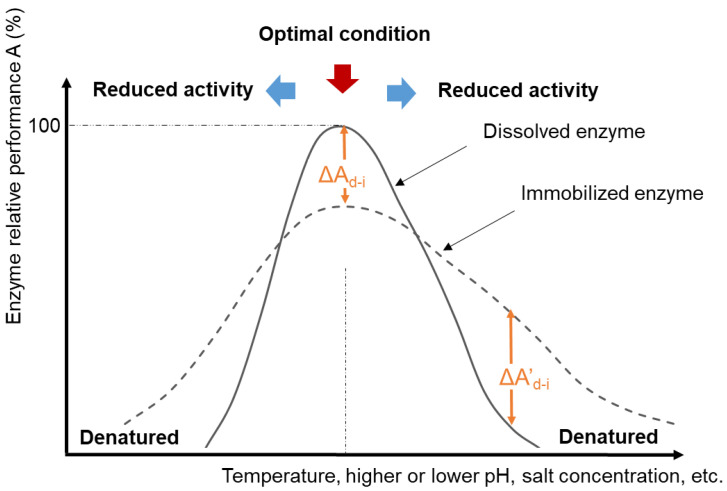
Schematic diagram of normalized enzyme activity and/or structural stability and process conditions for catalytic reactions.

Enzymes exhibit the fastest catalytic effects at certain “optimal” conditions of temperature, pH, ionic strength, and other factors (Figure 3). These conditions vary for specific isozymes and between enzyme classes. The optimal pH may be associated with the ionization state of functional groups within the active site when this is important for the catalytic mechanism. The optimal temperature often corresponds to the temperature just below the enzyme denaturation temperature because reaction rates generally increase as temperature increases. When enzymes denature, the protein structure unfolds to an extent that the three-dimensional orientation of the active site is disrupted, leading to a loss of catalytic activity. The optimal activity conditions may or may not correspond to the conditions at which the enzyme structure is most stable [44]. For example, a partially denatured (unfolded) enzyme might exhibit higher than usual catalytic activity (provided that the active site is still intact) because the active site may be more exposed to its substrate [45] and (especially if a higher temperature is a factor in the partial unfolding) the reaction kinetics will be faster [46]. Outside of the optimal activity zone, enzymes may still catalyze the reaction, but at reduced rates. Compared to dissolved enzymes, research shows that immobilized enzymes often demonstrate a higher tolerance toward more extreme process conditions [1,2,4,47,48,49] (dashed line in Figure 3). However, there can be trade-offs between enzyme stability (extended activity even under stressed conditions) and enzyme catalytic activity (the rate at which substrates are converted to products), which must be taken into consideration when comparing their overall catalytic efficiencies [44].

Protein engineering can create enzymes that are more robust than wild-type (those found in nature) isozymes through recombinant DNA techniques [50,51,52,53,54,55,56]. Another strategy for preserving enzyme activity is to hold enzymes (by immobilization) at relatively mild conditions to produce more stable biocatalyst products for industrial applications [57]. Immobilization also converts soluble enzymes to an insoluble form, making it readily separable from the process liquids during or after the catalytic cycle for recycling and to prevent enzyme contamination in products. In some cases, this separation prevents biocatalysts from being exposed to subsequent (harsher) process steps, thereby eliminating the risks of denaturation. Immobilization also makes it possible to install biocatalysts in continuous process flow reactions (such as packed-bed reactors), where extending enzyme performance longevity reduces enzyme consumption, resulting in higher overall productivity.

## 3. Quantifying Immobilized Enzyme Performance

**Figure 4:**
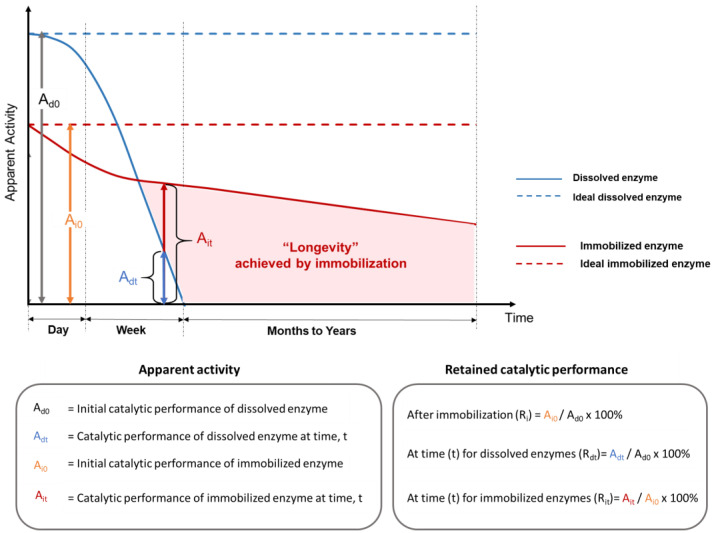
Relative enzyme performance versus process time for free and immobilized enzymes as justification for immobilizing enzymes to fabricate biocatalytic materials. R_i_ is the retained catalytic performance after immobilization (corresponding to activity recovery in Table 2). R_dt_ is the retained catalytic performance at a specified time for the dissolved enzyme. R_it_ is the retained catalytic performance at a specified time for the immobilized enzyme.

Measuring immobilized enzyme performance varies depending on which parameters are known and the purpose of the evaluation. Underlying these variabilities are the facts that different enzymes catalyze different chemical reactions at different rates with different optimal conditions, and immobilization techniques that work well for one enzyme type do not always translate well to others. Nevertheless, a number of metrics have become ‘expected’ (Table 2); however, these are not uniformly applied, causing comparison difficulties among published studies. The most important analytical parameter is enzyme activity, which is the enzyme-catalyzed reaction rate, often expressed in units of micromoles of substrate converted (or product generated) per minute. When the amount of enzyme protein is known, this value can be expressed as “specific activity”. However, the activity depends on many factors. As illustrated in Figure 4, even if reaction conditions are held constant, time and the impact of the immobilization itself influence the apparent enzyme activity. Other ways of quantifying immobilized enzyme performance are concerned with the catalyst consumption and conversion efficiency of the reaction as a basis for cost calculations. Productivity is an all-encompassing performance metric that is especially relevant for continuous reaction processes that are intended to operate for long periods of time (“longevity”). Due to its practical importance, longevity is explicitly called out as a measurement parameter in the productivity calculation shown in Table 2.

### 3.1. Retained Activity after Immobilization

When enzymes are immobilized, their detectable level of activity usually changes, resulting in an apparent (or relative) enzyme activity (abbreviated here as “A”, vertical axis in Figure 4). After immobilization, the measured activity is not only a consequence of specific enzyme activity and reaction conditions but also includes other factors, such as the mass fraction of immobilized enzymes in the support, the chemical or physical properties of the support materials, and the accessibility of enzymes in the immobilization matrix. The retained enzyme activity after immobilization can be quantified as A_i0_/A_d0_ (%), where A_i0_ and A_d0_ are the initial catalytic performance of immobilized and dissolved enzymes, respectively. For commercial processes, it is often essential that immobilized enzymes should have improved productivity (total amount of substrate converted per amount of enzyme protein) to generate sufficient cost savings to offset the extra cost of immobilization production and motivate adoption of the technology [60]. There are some exceptions to this, such as the production of high-value products that cannot be made by other methods and the use of enzymes for certain kinds of sensor design, in which the redox potential of the metal bound at the enzyme active site is more important than the chemical catalytic function of the correctly folded enzyme molecule [61]. Nevertheless, in most cases, extended enzyme catalytic longevity is indispensable, and the retention of enzyme structural stability is a prerequisite for enzyme activity.

It is not uncommon for immobilized enzymes, especially ones in retrievable solid matrices, such as membranes, to demonstrate lower activities at optimal conditions compared to that of the dissolved enzyme (A_i0_/A_d0_ < 100%). This lower activity is attributed to increased mass transfer barriers between substrates/products and enzymes in the presence of the immobilization matrix [18,62]. Conversely, many immobilized enzymes demonstrate better catalytic performance at broader pH or temperature ranges in comparison with dissolved enzymes, corresponding to a negative ∆A’_d-i_ (Figure 3) at conditions outside the optimal conditions for the soluble dissolved enzyme [10,63,64,65,66,67]. The exact reasons for improved thermal and pH stability through immobilization are not always clear, but the most common explanation is that the presence of the support provides confined environments to restrict the unfolding of enzymes when conditions become unfavorable. Studies also found that the presence of macromolecule crowding around the protein can tighten the protein structure and assist the protein folding [19].

### 3.2. Enzyme Activity over Extended Periods

Although a drop in the instantaneous enzyme performance between dissolved (A_d0_ in Figure 4) and immobilized enzymes with physical support (A_i0_ in Figure 4) is often observed at optimal reaction conditions for the soluble enzyme, the true benefit of using an immobilized enzyme emerges when the real application exposes the unprotected soluble enzymes to intolerable conditions, but spares the protected immobilized enzymes. A comparison of enzyme longevity for dissolved (A_d0_ and A_dt_) and immobilized (A_i0_ and A_it_) enzymes in terms of the apparent enzyme activity versus time is illustrated in Figure 4. A_dt_ and A_it_ are the catalytic performance of dissolved and immobilized enzymes at time t, respectively. The depicted rapid activity loss of soluble enzyme is often observed experimentally, either due to enzyme denaturation or to the difficulties of recovering and reusing dissolved enzyme proteins. This type of significant performance drop is indicated by a low A_dt_ over short periods (hours to days) in real applications (solid blue line in Figure 4), if no supplemental enzymes are added [68]. In comparison, the retained activity of enzymes that are bound to a physical support over very long time periods (months to years) is a hallmark of the improved enzyme longevity that can be obtained via immobilization (shaded area in Figure 4).

Over time, immobilized enzymes will eventually exhibit decreases (A_it_ < A_i0_) in relative performance (red curve) compared with an ideal scenario (A_i0_, red horizontal dashed line, Figure 4). Commonly, a first abrupt performance decrease is observed over a relatively short time period (e.g., hours to days), which is usually attributed to enzyme leaching from the support matrix. A slower biocatalyst inactivation or slower decrease in relative enzyme performance is then observed over a much longer period (e.g., weeks to years). A number of factors can contribute to this slower inactivation of immobilized enzymes, including gradual enzyme leaching, damage to immobilized enzymes, erosion or degradation of the physical support matrix, and accumulation of contaminants or fouling in/around the immobilized system. The magnitude of the retained activity difference between immobilized enzymes and dissolved enzymes (A_it_ − A_dt_) is determined by various parameters, such as inherent enzyme stability, support material properties, methods of immobilization, and application reaction conditions.

Therefore, immobilization not only provides more robust enzyme products against harsh catalytic conditions, but also provides high enzyme productivity that can enable commercial processes within the cost window allowed by the application [60], such as the continuous production of high-fructose corn syrup using glucose isomerase in the form of immobilized granules in large packed-bed columns [69]. Measuring biocatalytic longevity at lab scale may require special experimental setups for the targeted applications. For instance, lipase immobilized on cellulosic beads showed >700 h activity within a bench-scale reactor [70], and carbonic anhydrase immobilized on fibrous textile structured packing [71] retained 100% and 85% of the initial CO_2_ capture performance after a 71-day longevity test and after 1 year of ambient dry storage, respectively, tested using a lab-scale gas scrubber.

### 3.3. Mass Transfer and Surface Property Considerations

In enzyme-catalyzed reactions, the overall observed catalytic efficiency depends on: (1) the rates at which the substrate and product diffuse into and away from enzyme catalytic sites, and (2) the rate at which the substrate is converted to a product by enzyme molecules [72]. Enzyme immobilization can lead to changes in enzyme structural conformation, molecular steric hindrance, and changes in local charge density and pH near the surface that change the enzyme’s microenvironment and impact its activity [73,74]. Enzyme immobilization to a physical support can also change the accessibility of substrates as well as the diffusion of products, usually resulting in slower diffusion—referred to as ‘mass transfer (or mass transport) limitations’—due to a liquid boundary layer near solid surfaces that typically experiences lower turbulent flow and a lower concentration of reactants than the bulk liquid [75]. Mathematical treatments of mass transfer phenomena that incorporate both diffusion and reaction have been developed [76,77,78,79], and elaborated in detail, depending on specific reactor configurations, such as for porous gas–liquid hollow fiber membrane contactors, where an enhancement factor (E = V_catalyzed_/V_uncatalyzed_) can be incorporated to account for improvements in mass transfer due to the (bio-catalyzed) chemical reaction [80]. Simplified process metrics (namely “productivity”, Table 2) are reported when the detailed parameters needed for solving mathematical models are unknown [81]. Kinetic parameters impacting immobilized catalysts may change for multiple reasons, such as steric exclusion between support materials and enzyme substrates, delayed diffusion to interior pores of porous media, and changes in driving forces, such as concentration gradients induced by mixing or flow, that deliver substrates to or separate products from the catalytic matrix [74]. Models developed for immobilized enzymes with particulate form in packed-bed reactors [82,83] found that the smallest particle size that the reactor can handle, together with the highest enzyme loading on the particles, led to the best catalytic efficiency. Similar mass transfer variables and challenges occur for membrane reactors, where the requirement for flux through the membrane is an additional consideration that may require higher pore sizes (200–1400 nm) [84] than the 10–100 nm pore size range that tends to perform best with particulate biocatalysts [22]. Since reactive components and reaction configurations widely vary, fibrous membrane structures have strong potential to enhance substrate/product diffusion, because they have: tunable porosity and diversified surface chemistry (e.g., hydrophobicity, charges) for regulating enzyme loading, the potential to vary the curvature for decreasing denaturation and exposing enzymes to the reaction medium [84], and versatile hierarchical micro- and macroscopic sizes, shapes, and geometries that can physically create and support reactive surfaces while optimizing membrane spacing [75,85]. A recent study using single-particle analysis elucidated that the localization and packing density of immobilized enzymes also have significant impact on the kinetics of the catalytic matrix [86]. Such parameters can be controlled through various fiber formation techniques and immobilization approaches within membranes containing fibrous structures. Moreover, fibrous membrane physical flexibility and durability could enable unique reactor geometries that could even include self-standing catalytic reactors that facilitate the inlet and outlet of reactants and products.

**Figure 5:**
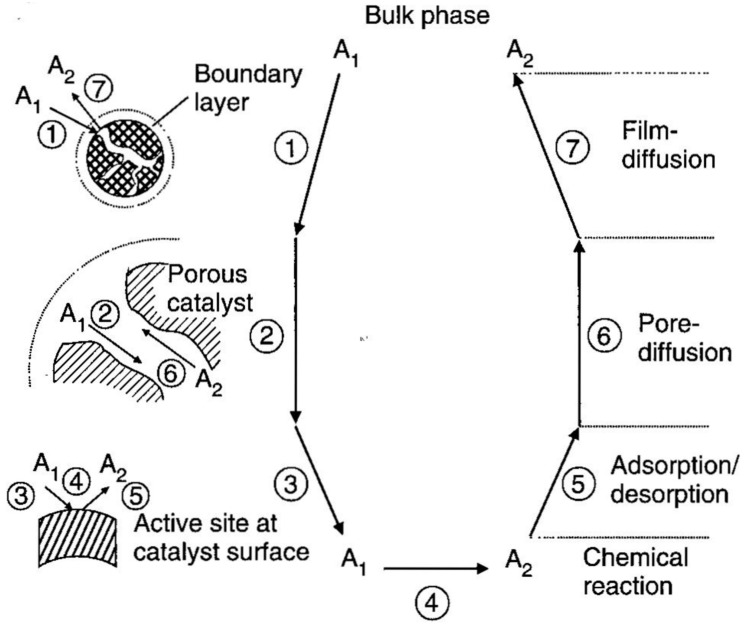
Individual steps of a heterogeneous solid–liquid catalytic reaction on a porous catalyst. A_1_ represents the substrate and A_2_ denotes the product (reprinted with permission from [87], Copyright 2008, John Wiley and Sons).

As heterogeneous catalysts, the mass transfer processes for membrane-bound enzymes can be described by theoretical frameworks developed for general heterogeneous catalysis [73,76]. The book chapter by Dittmeyer and Emig [87] illustrates the individual steps of a heterogeneous solid-liquid catalytic reaction on a porous catalyst (Figure 5). First, the substrate molecules (A_1_) in the bulk liquid phase need to diffuse through a stagnant liquid film close to the external surface of the catalyst. Then, the substrate molecules need to diffuse through the interior pores to reach the active site surface, where a series of adsorption, transformation, and desorption processes occur. Subsequently, the product molecules (A_2_) must diffuse back to the bulk liquid through pore and film diffusion. When the diffusion rate of the substrates from the bulk liquid to an immobilized enzyme’s active site is slower than the catalytic reaction rate, the observed rate, i.e., the apparent enzyme activity, is lower compared to the dissolved free enzyme. The rate of substrate flow in external mass transfer is often described by the product of a transport coefficient and the corresponding driving force, which is the gradient of the substrate concentration [76]. When a membrane is used, the external mass transfer is also proportional to the surface area [78].

The effectiveness factor (η = V/V_free_) ratio was introduced as an analytical solution to represent the change in the enzyme reaction rate upon immobilization. It can be calculated by measuring the kinetic parameters for free enzymes and immobilized enzymes [78,88,89]. This effectiveness factor is then used to determine the external mass transfer resistance for membrane immobilized enzymes and to determine the Nernst diffusion layer thickness [89]. The rate of internal mass transfer is considered to proceed in parallel with the enzymatic reactions [76]. Therefore, the change in the substrate conversion rate with immobilized enzymes is a sum of rate changes in diffusion and reaction inside membranes [76]. Therefore, geometric and chemo-physical properties, such as, pore arrangement, hydrophilicity, and pore sizes, significantly impact the overall mass transfer in the reactions [73].

A further dimensionless number, called Thiele modulus (∅), has been introduced to quantify the effect of the mass transfer limitation on the overall reaction [87]. It is defined as the square root of the ratio of the characteristic reaction rate in a bulk liquid phase over the effective diffusion rate at the external catalyst surface. In Equation (1), R is the radius (or thickness) of a typical porous pellet catalyst used for conventional reactors, k is the rate constant for an n-th order reaction, C_b_ is the concentration of the substrate in the bulk liquid, and D_e_ is the effective diffusion coefficient. Since the thickness of the catalyst layer on a membrane can be much thinner than typical pellet sizes, a Thiele modulus of as low as 1 is achievable in catalytic membrane reactors, signifying a complete utilization of the catalyst’s intrinsic activity [90]. Moreover, regardless of what physical forms are used in immobilization, appropriate reactor selection and design for these heterogeneous catalysts characteristically helps to enhance the mass transfer rate of the system [73].

(1) $$\emptyset =R\sqrt{\frac{kC_{b}^{n-1}}{D_{e}}}$$

## 4. Immobilization Chemistry

Fundamentally, immobilizing enzymes on physical supports [91] enables recycling enzymes in applications involving liquids [92]. Numerous prior reviews describe the evolution of enzyme immobilization over the years [1,2,92,93,94,95,96,97,98,99] and books provide diverse immobilization protocols [100]. As summarized by Zdarta et al., the main attributes of enzyme immobilization support materials include stability, insolubility, high affinity to enzymes, biocompatibility, availability, reusability, and the presence of reactive functional groups [30]. The composition of support materials ranges from inorganic substrates such as glass and carbon to advanced nanoscale composites and flexible soft matter [93,97,98,99,101,102,103].

High affinity between support materials and enzymes occurs through intermolecular interactions between enzymes and reactive chemical functional groups in the physical supports. Both native [7,8] and chemically introduced [37,48,81,104,105,106] amine groups (–NH_2_) are frequently used to physically or chemically attach enzyme molecules for immobilization purposes. Bagheri et al. achieved robust immobilization by utilizing spherical dendrimers with multiple amine end groups to covalently attach enzymes to a film surface [36]. Oxygen-containing functional groups, such as hydroxyl (–OH) and carbonyl groups (C=O), have been used for direct enzyme immobilization [32,74], as well as primers to introduce other chemical groups for immobilization [30,48,81,98]. Methods such as oxidation [30,48], chemical treatment [31,32], or plasma activation [106] are used to activate hydroxyl groups. Hydroxyl groups have also been used as crosslinking sites inside gel matrices to prevent leaching of entrapped enzymes [50]. Thiol groups (–SH) are another commonly used reactive group for enzyme post-immobilization. The interaction between the thiol groups from cysteine and gold nanoparticles forms enzyme–gold conjugates in sensor development [40]. Since the quantity of cysteine is much lower than amine groups in enzyme structures, the use of thiol groups enables site-specific immobilization, giving control over the orientation of immobilized enzymes [107].

Adsorbing enzymes onto the fiber surface through weak interactions, such as Van der Waal (VDW) forces, electrostatic attractions, hydrogen bonds, etc., is a straightforward post-immobilization approach. These mild interactions are known for their advantages in preserving enzyme activity by maintaining the structural flexibility of immobilized enzymes [1,29,98,108]. Reversible adsorption also has advantages in designing therapeutic enzyme delivery systems, from which the enzyme molecules can be released into the medium in a controlled manner (e.g., pH- or salt concentration-responsive). Notably, pH has been used in most post-immobilization cases as a tunable parameter for achieving desired enzyme loadings [63], adsorption rates [17], and/or the orientations of enzyme molecules on surfaces after immobilization [9,109]. Enzyme solution pH affects the protonation/deprotonation of amino acid side chains as well as the overall charge of the enzyme molecule, which are responsible for the interaction between the enzyme and the support.

Covalent attachment is another “standard” method for post-immobilizing enzymes because it forms durable linkages between the enzyme and the support [110]. Functional groups in enzyme molecules, usually the reactive groups on amino acid side chains, react with functional groups or moieties on support materials to form covalent bonds. Enzymes can be attached in the presence of catalysts and at suitable conditions to support materials with inherent reactive functional groups, while chemical pre-functionalization, plasma treatment, or radiation treatment are used to introduce necessary reactive groups on non-reactive supports [111]. Early research found that direct covalent interactions between enzymes and the support can cause undesired conformational changes in enzymes, leading to a large ∆A_d-i_ [1,2,112]. Further study showed that the distance between covalently immobilized enzyme molecules and the support surface is critical for enzyme structural stability [1,113]. Research on controlling the spatial distance between the enzyme and the support surface using crosslinking agents of different lengths identifies glutaraldehyde (GA) as the most commonly used crosslinker [2,48,104,105,106,114]. However, the presence of GA can cause enzyme inactivation throughout the immobilization [18,115]. Therefore, the ratio of GA to enzyme must be controlled, and kept at a low enough level to avoid excessive enzyme inactivation. Recently, Braham et al. [17] elucidated the mechanism of using GA to enable an energetically unfavored immobilization system with laccase, where the adjustment of ionic strength plays critical roles in enzyme stability and activity [17]. Other crosslinking agents with desired functional groups, such as silanes and cyanogen bromide, have also been applied for enzyme immobilization based on the chemistry of the supports [11,13]. The same types of immobilization chemistry can be used across different physical forms of immobilization supports.

## 5. Non-Fibrous Immobilization Supports

Granular solid physical supports have been widely explored due to their high surface area and ease of handling for packed-bed and batch reactors [116,117,118,119]. Two approaches, minimizing the particle size [120,121,122,123] and introducing porosity inside the particles [124,125], are often pursued to improve the performance of granular materials. This led to some general correlations between pore size and enzyme loading (loading favored for pore diameters in the 10–100 nm range) based on rigorous analysis of published data by Bayne et al. [22], but no clear correlations were found relating pore size to enzyme activity due to the diversity of the studies. Exceptions to general findings also abound. For example, when the substrates are macromolecules, better catalytic reaction efficiency may be observed with higher pore sizes (>1000 nm) and lower enzyme loading (1–3 mg/g matrix), associated with higher permeability and less molecular crowding, as was reported for the flow-through degradation of RNA by Ribonuclease A immobilized in macro-porous monolithic columns, with controlled pore sizes in the range of 360–2020 nm [126]. Alternatively, a hierarchical granular composite consisting of gold nanoparticles (1–3 nm diameter) that were covalently bound to mesoporous silica particles contributed to high carbonic anhydrase enzyme loadings (100–300 mg/g matrix) and extended enzyme longevity (98% activity retained after 20 days in pH 6.4 buffer at 25 °C) [125]. Enzymes immobilized on metallic nanoparticles have also shown larger ∆A’_d-i_ toward temperature and pH variations [120,121,122,123]. Silica-based granular supports have been used for either post-immobilization or enzyme incorporation during sol-gel fabrication [65,124,127,128,129,130]. Nest-like aluminosilicate-based microspheres functionalized with dopamine exhibited especially high laccase enzyme loadings (>300 mg/g matrix) due to their hierarchical porous structure and abundant chemical functionality [131]. For comparison, lipase enzyme loadings of 140 mg/g matrix were achieved with an epoxy-functionalized macro-porous (22 nm average pore size) poly(methyl methacrylate) resin, with a 230 m^2^ g^−1^ surface area [132]. Polymeric microbeads [104,133] and macroscale beads [67] are often used to take advantage of abundant functional groups and the convenience of the materials [134,135]. Carbohydrate polymeric beads formed at mild conditions from aqueous solutions, through mineralization or coagulation, offer good biocompatibility, along with improved thermal stability and activity retention of over 50% after several catalytic cycles [134,136,137,138,139,140,141,142]. Granular beads are good choices as immobilization supports in applications where the reactor system is well-adapted to the particle shape and physical properties (e.g., hardness). However, in some applications, such as packed-bed reactor configurations, small particle size can lead to an excessive pressure drop [74], and alternative immobilization support structures are needed.

Enzyme immobilization on two-dimensional nanomaterials is important for the development of bioactive electrochemical devices, such as biofuel cells and sensors [9,14,30,32,62,108,143,144]. Honeycomb-like graphene and its derivatives offer large surface areas (>1000 m^2^ g^−1^) for enzyme immobilization [32], and the combination of π–π bond stacking and abundant nucleophilic groups on the surface of oxygenated graphene (GO) facilitates interactions between the enzyme and the support, yielding high enzyme loading (~100 mg/g GO) [145], that had even higher loading levels (>300 mg/g matrix) when carbon nanotubes were incorporated to produce 3D “nanoflower” structures [146]. Graphene oxide-based catalytic materials also exhibit enhanced thermal stability and solvent tolerance via increased substrate conversion rates (a negative ∆A_d-i_) [32,143,144,147,148]. Producing these two-dimensional supports typically requires concentrated acids or thermal reduction, which would inactivate most enzymes. Therefore, enzymes are post-immobilized to the support from mild pH buffers [62].

Three-dimensional physical supports, such as films [18,115,129,149,150], membranes [12,16,63,151,152,153,154,155,156,157], and gel networks [8,10,158,159,160,161,162], can be fabricated as modular components of continuous reactors and offer robustness for enzyme immobilization as retrievable catalytic matrices. Although relatively high productivity can be obtained using these physical supports, due to enzyme reusability, a lower A_i0_ is often observed due to challenges in obtaining a high mass fraction of effectively loaded enzymes and mass transfer barriers in substrate/product diffusion [17,163]. To resolve this problem, fibrous membranes with ultrafine fiber structures have been extensively investigated as enzyme immobilization supports [12,156,164,165,166,167]. Major advantages of using fibrous materials as the support are the increased surface area and material flexibility. These physical properties are well-suited to continuous-flow reactors and applications requiring specialized geometries. Due to their flexible physical form, fibrous membranes can be installed in continuous reactors in ways that minimize pressure drop and enable positioning the biocatalysts in unique and useful ways. Their large, exposed surface area is expected to contribute to high enzyme loading and efficient mass transfer. Fibrous membranes by themselves are already used for many applications, including water purification, air filtration, wound dressings, and therapeutic implants; therefore, the additional biocatalytic functionality introduced by enzyme immobilization offers many opportunities to expand the utilization of these fibrous materials [155,168].

## 6. Fibrous Membrane Immobilization Supports

Fabricating fibrous membrane materials with scale-up potential can be carried out using conventional or advanced textiles and nonwoven industrial equipment. A plethora of fiber-forming precursors offer diverse fabrication options, such as inorganic fibers, synthetic polymeric fibers, fibers from natural resources, and fibers produced from polymer blends [7,99,122,166,169,170,171,172,173,174,175,176,177,178,179,180,181]. Processing parameters in fiber formation govern the chemo-physical properties and structural features of the fibrous products, offering a huge innovation space to enhance immobilized enzyme properties. Advanced fiber formation techniques, such as electrospinning, are capable of producing nanoscale-diameter fibers with unique properties that have promoted their use for enzyme immobilization [152,153,171,173,182,183,184,185,186]. Depending on the fiber-formation process, enzymes can be immobilized by three basic approaches: (1) direct enzyme post-immobilization on fiber surfaces after fibrous membrane formation, (2) enzyme incorporation inside fibers during membrane formation, and (3) hybrid methods, where enzymes are adhered to fibrous membrane surfaces using coating techniques. An extensive summary of recent enzyme immobilization studies on fibrous membranes is provided in Table S1 of the Supplementary Materials. Table 3 summarizes selected studies in which quantitative measures of immobilized enzyme properties and performance were presented; however, standard metrics, such as enzyme loading, are inconsistently reported. Key features and noteworthy examples representing the three basic immobilization techniques for fibrous membranes are emphasized in the discussion below.

### 6.1. Post-Immobilization on Fibrous Membranes: Enzyme Immobilization after Fiber Formation

Similar to other forms of physical support, enzymes can be immobilized onto pre-formed fibrous membranes through either covalent or non-covalent interactions. Thanks to post-immobilization methods, fibers obtained from high temperatures (e.g., melt extrusion) are able to immobilize thermally unstable enzymes, and enzymes with low tolerance to solvents can be post-immobilized to fibers made from organic solvents. Membranes containing nanoscale fibrous features are gaining popularity as immobilization supports [16,62,99,108,121,122,139,153,154,171,172,197,198,199].

Lipase is a frequently used model enzyme for demonstrating novel immobilization methods. For example, physically adsorbed lipase on commercial inorganic glass fibers and carbon fibers was used for gas-phase hydrolysis and transesterification [169]. In this case, the hydrophobicity of the fibrous membrane support had a positive impact on the catalytic efficiency [169]. To catalyze hydrolysis reactions in a liquid phase using immobilized enzymes, lipase was covalently attached to polymeric electrospun webs [187]. Recently, Liu et al. utilized feather polypeptides as an enzyme-protecting agent in preparing fibrous supports for lipase post-covalent immobilizations, from which enhanced enzyme thermal stability (larger ∆A’_d-i_ toward temperature variation) was reported [153]. Moreover, by using electrospun collagen fibers that contain magnetic particles to post-immobilize lipase, enzymes retained activity at broader pH ranges (larger ∆A’_d-i_ toward pH variation) and were recycled by applying a magnetic field [166]. In addition to the inorganic fibers, synthetic fibers, and protein fibers, lipase has also been post-attached to modified cellulose fibers [113].

Other enzymes, such as glucose oxidase, laccase, carbonic anhydrase, and peroxidase, have been post-immobilized onto fibrous matrices to obtain functional materials, such as hemostatic wound dressing materials [200,201], antimicrobial surfaces [188,202], adhesion-reduced implants [12,157], and gas scrubbing membranes [203,204]. A silk fibroin, nonwoven, was used as the support for post-immobilizing glucose oxidase in the fabrication of flexible glucose sensors [10]. Amine-functionalized woven polylactic acid (PLA) was used to post-immobilize trypsin with the assistance of glutaraldehyde, after which improved trypsin thermal and pH stability and reusability of the enzyme were reported, for at least 15 catalytic cycles [189]. Urease has been immobilized onto an as-spun polyacrylonitrile (PAN) fibrous membrane from phosphate buffer, forming bioactive urea hydrolysis material with reusability [184]. A post-immobilized tyrosinase on a polycaprolactone (PCL)-chitosan composite showed enhanced longevity after storage, while its durability in a continuous catalytic reaction was reported up to 2 h [205]. Recently, traditional textiles, such as yarns and fabrics, are gaining attention as enzyme immobilization supports, due to their intrinsic hierarchical structures that offer diverse fabrication options [71,206,207]. Wool fabrics (protein-based) were used to immobilize lysozyme to fabricate antibacterial fabric [188] and to study the compatibility between the enzyme and the support [208]. To graft biomolecules onto wool fibers, enzymatic reactions using protease were applied as an activation step ahead of immobilization [209]. As the most abundant renewable polymer on earth, cellulose-based materials are promising candidates for post-immobilizing enzymes, including cellulose nanocrystals [64], bacterial cellulose [139,202], regenerated cellulosic materials [113,210], cellulosic paper [211], and cellulosic fabrics [212]. For this group of materials, post-immobilization methods are preferred. Other than using the intrinsic hydroxyl group on cellulosic fibrous materials directly, acetyl groups in cellulose acetate fibrous membranes were hydrolyzed to hydroxyl groups for grafting lipase [113]. Arola and co-workers utilized epoxy-, amine-, and carboxylic-functionalized cellulose to attach alkaline phosphatase, resulting in enhanced enzyme stability [64]. Recently, Böhm et al. reported a prototype of a chromatic paper-based microfluidic sensor with glucose oxidase and peroxidase, from which efficient glucose detection was demonstrated through an enzymatic cascade reaction [211]. Post-immobilization approaches with covalent linkages usually come with sophisticated fabrication steps and numerous chemical reactions are often required, including toxic and high-cost chemicals. Therefore, gaps still exist between laboratory prototypes and commercialized products with scale-up potential for industrial applications.

### 6.2. Incoporation in Fibrous Membranes: Enzyme Immobilization during Material Formation

Compared to post-immobilization approaches, incorporation immobilization can achieve high mass fractions of enzymes using simple procedures [110]. In this strategy, enzyme immobilization occurs simultaneously during support material formation [213]. Thus, either a mild fiber fabrication procedure or a robust enzyme is required, which restricts the selection of raw materials. Chemical functional groups on the supports are not essential for immobilization, but they can either be used to facilitate the immobilization procedure through enzyme–matrix interactions, or to adjust the porosity of the fibrous membrane [8,67,136,214].

Diverse forms of silica or silica-based particles synthesized by the sol-gel method [8,127,160] or water-soluble polymers [29,67,136,137,162,171,214] are commonly used as enzyme incorporation matrices. These polymers have the potential for being processed into fibrous membranes [98,172,173,187]. Among existing membrane supports with enzymes incorporated, enhanced thermal or pH stabilities (larger negative ∆A’_d-i_ toward temperature or pH variation of enzymes) were reported [18,66,70,127,169,215,216]. At the same time, the incorporation method resulted in a lower relative enzyme activity (larger positive ∆A_d-i_) compared to free enzymes and/or enzymes post-immobilized on fiber surfaces, due to the added mass transfer barrier for entrapped enzymes, as described in Section 3.3. However, because the procedure is straightforward, when adequate performance is achieved, enzyme incorporation is an advantageous method for easy scale-up.

A flexible sensor, fabricated by coating glucose oxidase, entrapped in a structural protein network, onto a flexible nonwoven material, exhibited improved stability to pH fluctuation [10]. When water-soluble synthetic polymers that dissolve at mild conditions, such as polyvinyl alcohol (PVA) and polyethylene oxide (PEO), are used for incorporating enzymes during fiber formation, the process can be followed by additional crosslinking steps to tune the solubility of the resulting matrices comprising enzymes [151,172]. For example, α-amylase incorporated in electrospun PVA fibers retained around three times the catalytic activity at 80 °C compared to free enzymes [195]. Emulsion methods have been used to incorporate α-amylase in electrospun ethyl cellulose fibrous membranes, with entrapped enzymes showing improved stability after 45 days of storage, and catalytic membrane activity retention of 100% and 50% after 10 and 15 catalytic cycles, respectively [196]. When laccase enzymes were entrapped together with γ-cyclodextrin or as pre-made enzyme-cyclodextrin inclusion compounds that were immobilized in poly(ε-caprolactone) (PCL) nanofibers from organic solvents, the specific activity of immobilized laccase increased by 3- to 9-fold compared with that of directly entrapped bare laccase exposed to organic solvents during PCL fiber formation [197].

The strong acidic or alkaline conditions required for solution processing of cellulosic materials are not favorable for direct enzyme entrapment. Emerging research in ionic liquids (ILs), including their application in polysaccharide processing [217,218,219] and potential for enzyme immobilization [18,70,216], provides new opportunities to entrap enzymes into cellulosic-based fibrous materials during material formation. Peroxidase extracted from horseradish root survived an alkaline coagulation solution and was successfully entrapped into chitosan beads, showing higher stability in a dye decolorization process [137]. Since the bioactive mixture from a plant extract was used for immobilization, rather than purified enzymes, the stability-enhancing mechanism was unclear. Other strategies, such as using glycerol, have been reported to mediate the mixing of enzymes with an acidic chitosan solution during lysosome incorporation [220]. Positively charged chitosan was coated onto enzymes entrapped in negatively charged alginate beads through electrostatic interactions of these two oppositely charged polyelectrolytes, which improved enzyme thermal and pH stabilities and longevity [138]. A limitation of the incorporation method through reversible interactions is the undesired low enzyme longevity caused by gradual enzyme leaching in a liquid medium. To overcome this problem, crosslinking agents are used to reduce the pore size of the gel network where enzymes are entrapped [67,134,158,159,170]. In addition to the commonly used chemical crosslinker, GA, fusion proteins can enhance enzyme incorporation in cellulosic materials (e.g., via carbohydrate-binding domains) [221].

### 6.3. Hybrid Methods for Fibrous Membranes: Biocatalytic Coatings on Structrual Supports

The foregoing immobilization methods each have advantages and disadvantages for fabricating efficient and robust biocatalytic fibrous matrices. Post-immobilization offers infinite flexibility in the selection of matrix materials and exposes enzymes at surfaces to minimize mass transfer barriers, but may require complex covalent attachment protocols, while direct enzyme incorporation during matrix production can offer simple ‘one-step’ fabrication and ‘protect’ enzymes within the matrix, but has more limitations on the choice of support material and processing conditions, and may increase mass transfer limitations by ‘burying’ enzymes within the matrix. Hybrid coating methods can exaggerate all the advantages, while minimizing the disadvantages. For example, a thin layer of gel comprising enzymes was coated onto a prefabricated textile support to reduce mass transfer barriers [10]. The resulting biocatalytic material yielded a higher glucose sensing output signal, compared to the enzyme entrapped in a thick gel membrane, thanks to the increased surface area of the fibrous support, which improved glucose diffusion in the thin layer [10]. This result supports the concept of creating high surface area thin coatings to overcome mass transfer barriers, while providing a protective environment and higher enzyme loading using hybrid methods. In another example, a biosensing film made of a metal-organic framework containing glucose oxidase was coated onto an optical fiber for a 1–8 mM glucose sensing range, with a good response coefficient of ~0.5 nm/mM [222]. In another glucose sensing study, an optical fiber was coated with a cross-linked electrospun fiber containing enzymes and an improved sensitivity of 1.875 dB/mg·mL^−1^ was reported [223].

According to the literature, most immobilizations of biomolecules on textiles are for biomedical applications (e.g., wound dressings), rather than considering the advantages of using textile materials as immobilization supports for industrial applications. Enzymes and immobilized enzymes have been used in the textile industry for decades [1,224,225], as sustainable solutions to reduce chemical consumption in apparel and textile manufacturing and to treat the textile process effluents. However, using textile materials as immobilization supports, and applying novel textile technologies to enzyme immobilization procedures, is a rather new research field [226,227]. A study reported remarkable potential for using textiles (fabrics) in fabricating recyclable metallic catalysts, which could inspire broader utilization of traditional textile supports [226]. More recently, chitosan was used together with catalase or carbonic anhydrase to form thin (<0.5 µm) catalytic coatings on cellulosic textiles [206,207]. The opposite charges between chitosan and cellulosic fibrous supports provided relatively strong bonding, while the mild incorporation immobilization approach preserved enzyme activity. Resulting biocatalytic textiles were used to improve the reaction efficiency of three-phase (gas-liquid-solid) catalyzed reactions by minimizing diffusion limitations. In the catalase example, with a flow-through configuration, the biocatalytic fibrous material decomposed at least two times more peroxide in a twenty-times smaller reaction zone volume compared to a stirred tank configuration [207]. Further innovations in hybrid biocatalytic material fabrication are possible. For instance, a textile (e.g., fabric, yarn) can act as a biocatalytic reactor to carry catalytic substrate/products to a designated immobilized enzyme through the wicking of liquids that carry the substrate/products. Since the wicking profile of textiles or textile-membrane hybrids is governed by its composites and structures, biocatalytic reactors with controllable reaction rates can be designed based on controlling these parameters.

### 6.4. Polymer Selection in Fibrous Membrane Supports for Enzyme Immobilization

Polymers are extensively used to produce diverse fibers and coatings that are equally attractive for fabricating fibrous membrane supports for biocatalysts. The advantages of using synthetic polymers for enzyme immobilization [98,155,167,173,174,183,184,228,229] are the abundant functional groups possible in their structures, the controlled molecular architecture, and processability [1,30,230], with versatile fabrication possibilities, such as production of hollow fiber membranes [231,232]. The intrinsic properties (e.g., solubility, polarity, hydrophobicity, rheology) of synthetic polymers influence the immobilization procedures [133] and can be modified by crosslinking to facilitate post-immobilization procedures [18,110,197]. Some synthetic polymers are widely available and relatively inexpensive, while polymers with specialized chemical functionality or physical properties can be more costly.

Increasingly, polymers from renewable sources are attracting research interest as sustainable alternatives to petroleum-based chemicals in many emerging research areas, including enzyme immobilization, particularly for fibrous membrane supports [10,64,139,153,233]. Along with their biobased origins, natural polymers tend to have abundant chemical functionality, which gives them high affinity and compatibility with enzyme protein molecules. Biobased, biodegradable, synthetic polymers, such as PLA, have been used as immobilization supports for specialized applications, such as the delivery of reversibly immobilized protein molecules to targeted locations or release of encapsulated enzymes into a medium through gradual degradation of the matrix [201]. For example, encapsulated superoxide dismutase (SOD) and catalase (CAT) were used to deliver antioxidants in vivo from microspheres made of poly(D,L-lactide-co-glycolide) (PLGA) and poly(D,L-lactide) (PLA) [133].

Polysaccharides, such as cellulosic materials, chitosan and chitin, alginate, and agarose, have been applied as biobased supports for immobilizing enzymes using both post-immobilization and incorporation methods. For instance, agarose obtained from seaweed was used to produce highly porous and mechanically resilient matrices for enzyme immobilization, where the matrix pore size was adjusted by the agarose concentration and crosslinking agents [30,234]. Alginate solutions form gel networks when cations (usually divalent calcium) are present [136,214]. Post-immobilized lipase on an alginate-polymer membrane exhibited enhanced thermal and pH stability and a 50% retained relative enzyme performance after 14 catalytic cycles [171]. A reverse hybrid construction was carried out by culturing bacterial cellulose to coat the outside surface of sodium alginate beads, followed by adsorption to immobilize lipase on the fibrous coating [139]. Such immobilized enzyme hybrid strategies overcome the fragile nature of alginate to maintain hydrogel properties, while improving the mechanical strength [136,139,235]. Natural fibrous sponge, *Luffa cylindrica*, was used as a biobased scaffold for laccase immobilization, and a negative ∆A’_d-i_ toward pH and temperature variation was reported [236]. Natural fibrous membranes generated from pulp and paper waste streams were used in laccase immobilization for repeated degradation of the pharmaceutical contaminant sulfamethoxazole [237] or dye decolorization [67]. Membranes made of structural proteins have also been used to immobilize glycerol dehydrogenase and diaphorase for triglyceride detection from serum at relatively low concentration ranges in a short time [238]. Polypeptide chains were used as additives to stabilize a covalently immobilized enzyme on polymeric fibers and improved catalytic performance was observed with lower enzyme loadings [153]. Silk fibroin has also been used to stabilize entrapped enzymes in developing novel materials and devices [10,150].

## 7. Characterization of Enzyme-Immobilized Fibrous Materials

Once enzymes are immobilized onto or inside a fibrous support material, the overall catalytic performance depends on many factors, including the total available enzymes, the physical properties of the fibrous support materials, and the interaction between the enzymes and the support. These complex systems require multiple characterization methods to elucidate their structures and functional mechanisms.

### 7.1. Fibrous Support Materials

Characterizing the non-catalytic properties of support materials can be carried out using a wide range of material science characterization methods. In general, chemical compositions of newly synthesized or treated materials are confirmed by spectroscopic methods such as FTIR, Raman, and NMR [64,229], and by known characteristic physical properties such as crystal structure (XRD), melting temperature (DSC), and decomposition (TGA) temperature [113,239,240,241]. For covalent attachment immobilization, the surface density of functional groups, such as free amines, free carboxylic acids, and free aldehydes, plays a vital role, and can be determined by titration [151], colorimetric assay [12], or elemental analysis [200]. For surface adsorption immobilization that leverages ionic interactions, the surface charge density can be characterized by zeta potential measurement [242] or a simple color depth observation from the adsorption of oppositely charged dye molecules [208]. The hydrophilicity or wettability of a surface, as manifested by differences in the water contact angle [189], which is extremely sensitive to surface chemistry and morphology, not only dictates the interaction between the enzyme and the support [2], but also determines the transport properties of substrates going into and out of enzyme active sites [155].

More work is still needed to build a deeper understanding of structure–property relationships between material structural parameters and the mechanistic performance of immobilized enzymes to promote future developments. This includes emerging interest in determining how fibrous materials potentially participate in the active transport of process liquids to and from enzyme active sites, beyond merely providing contacting surfaces for the reaction, such as a case where liquid traveling within a textile yarn instead of on and over it was observed [207]. Table 4 lists the characteristic parameters that should be considered when developing fibrous immobilization matrices. Several are unique to fibrous materials.

For man-made fibrous materials, both biobased and petroleum-based, the fiber diameter is tunable through the choice of spinning techniques and associated adjustable parameters. In principle, measurement of the fiber diameter is straightforward and can be carried out using optical or electron microscopes (POM, SEM, TEM, etc.). A smaller fiber diameter increases the surface area, which is often associated with higher enzyme loading [243] and better storage and operational stabilities of immobilized enzymes [244]. The specific surface area (area per mass) of non-porous cylindrical-shaped fibers, which is a good approximation for many synthetic fibers, can be estimated from the bulk density of the polymer material and the fiber diameter through simple geometry [245]. Nitrogen adsorption–desorption isotherms, which measure the adsorbed gas volume versus pressure at a constant temperature, are used to calculate the surface area along with the pore volume and pore size using theoretical models [163,166,243]. A capillary flow porometer is used to obtain the pore size and pore size distribution for interconnected pores in filtration and flow-through devices [155,246]. The principle of this technique is based on gradually increasing the upstream gas pressure while monitoring the increase in gas flow as it pushes through a sample that was completely wetted prior to the measurement by capillary forces. Thus, larger pores are emptied first, followed by smaller pores, until the gas flow rate overlaps with that of the dry control sample. In addition, relevant application parameters such as liquid entry pressure (LEP) [163] and clean water permeance [246], according to standard testing protocols, are adopted by the applicable industry. Currently, surface area measurements are not routinely presented in immobilized enzyme publications; however, several examples pertaining to enzymes immobilized on fibrous materials are summarized in Table 5.

### 7.2. Immobilization Effectiveness and the Performance of the Immobilized Enzymes

To evaluate the quality of an immobilization process, the amount of enzyme that becomes immobilized and its activity must be determined. Despite different terminologies used in the literature, immobilization success is basically assessed in the form of percentages, calculated by dividing the two aforementioned quantities by the known total starting enzyme amount (immobilization yield) or the total starting enzyme activity (activity recovery). The amount of enzyme (or activity) that is immobilized is determined by subtracting the residual protein amount (or activity) in the supernatant (plus any rinsing solutions) from the starting protein amount (or activity) [169]. Metrics based on enzyme activity units are preferred when non-purified enzyme products (containing mixtures of other non-active proteins) are used for immobilization, due to the different immobilization yields of different proteins [58].

A related quantity that also characterizes the amount of enzyme immobilized is the enzyme loading. It differs in the calculation denominator, where the weight of the support material instead of the total starting enzyme amount is used. The percent immobilization yield and activity recovery only evaluate the efficiency of the immobilization process and can sometimes be misleading, as a higher yield does not necessarily lead to higher observed immobilized enzyme activity. Since the enzyme loading metric is normalized by the weight of the support material, which is independent of immobilization conditions, this value allows comparisons to be made between different support materials.

Central to the evaluations discussed above is determination of the protein concentration. The most commonly used methods are protein detection assays, including the Lowry [248] and Bradford methods [165,245,249]. It is also possible to measure the protein concentration using absorption at 280 nm on a UV-VIS spectrophotometer [250]. However, measuring residual protein concentration in the supernatant is an indirect way of estimating the amount of immobilized enzyme, which often induces experimental errors because enzymes precipitated during the immobilization process are not detectable in the measurement, resulting in overestimation of the amount immobilized. In cases where metalloenzymes are used, enzyme loading can be obtained directly from the immobilized enzyme on the support. Opwis et al. demonstrated the use of atomic absorption spectroscopy (AAS) for obtaining catalase loading on cotton fabrics using various immobilization methods [251]. Essentially, the enzyme-immobilized support was “digested” in the chemical pretreatment using concentrated sulfuric acid and hydrogen peroxide, and “burnt” in the flame of an atomic absorption spectrometer using an acetylene–air mixture as the burner gas. The iron content of the sample was compared against that of a catalase standard curve to yield catalase loading per sample. In another example, Wunschik et al. used inductively coupled plasma optical emission spectroscopy (ICP-OES) to obtain the amount of peroxidase immobilized on polyester felt fabric, specifically comparing the iron content of the immobilized enzyme sample against a calibration curve made with the commercial peroxidase being used and a blank made with surface-functionalized textile support without enzymes [252]. Sample preparation for the ICP-OES measurement also involves a chemical decomposition step where, in this case, 69% nitric acid and microwave heating were used to break down the enzyme-immobilized textile sample. In a different situation, where the support material has a very different thermal decomposition profile than the enzyme, the approximate enzyme loading can be obtained through thermogravimetric analysis (TGA). Mohamad et al. immobilized a significant amount of lipase onto acid-functionalized multi-walled carbon nanotubes (F-MWCNT) by physical adsorption, and an enzyme loading of 13% was deduced by comparing the thermal decomposition profiles of the no-enzyme F-MWCNT and the enzyme-immobilized MWCNT [253]. A comparison of the sample requirement, detection limit, and operation principle of these direct enzyme loading detection methods is shown in Table 6. In addition, a nitrogen content measurement from either combustion or wet chemistry methods can be used to estimate enzyme/protein loading on the support owing to the high nitrogen content of protein compared to the support materials [242,254]. When precise measurement of enzyme loading per unit area is needed, a quartz crystal microbalance can be used to measure extremely small mass increases caused by the deposition of enzyme layers on model surfaces prepared directly on the quartz crystal [64,130].

Enzyme activity assays are used to evaluate both the quality of the immobilization process and the performance of the immobilized enzymes [153,183,184,255,256]. Detection of decreased activity after samples are incubated at elevated temperatures can be used to fit thermal deactivation models and calculate the thermodynamic parameters of the deactivation process [247]. By varying substrate concentrations in the enzyme activity assay, kinetic parameters can be calculated from fitting the Michaelis-Menten kinetic model [152,257]. Enzyme activity assays are also used to evaluate immobilized enzyme storage stability over time and reusability after repeated cycles of use [156,167,187].

While most enzyme activity assays are conducted as batch reactions (often in small vials or microwell plates), the ultimate evaluation of immobilized enzyme performance needs to be carried out in real application configurations. Nair et al. devised a continuous-flow reactor to measure the steady-state hydrolysis of 4-nitrophenyl butyrate by lipase immobilized on polystyrene-based nanofibers, and the apparent rate constant of the reactor as a whole [250]. Ibrahim et al. tested antimicrobial properties of a cotton fabric immobilized with a combination of α-amylase, alkaline pectinase, and laccase against bacteria and fungi over 30 laundry cycles for potential biomedical applications [258]. Similarly, Coradi et al. immobilized pectinolytic enzyme alkaline pectinase on cotton fabric and found good antibacterial activities [259]. Park et al. evaluated the antibacterial properties of lysozyme-CLEA-immobilized chitosan nanofibers against four bacterial pathogens for 10 cycles [260].

Enzymes which catalyze reactions involving the transfer of electrons can be characterized by electrochemical tests in their respective applications. Fu et al. immobilized laccase on a cellulose nanofiber/silver nanoparticle composite as a biosensor for the detection of catechol, and cyclic voltametric measurement was used to monitor the electrochemical activity in the presence of the substrate [122]. Kim et al. evaluated the maximum power density of the biofuel cell composed of an enzyme anode fabricated with glucose oxidase immobilized on polyaniline nanofiber matrix in the presence of glucose and air [243]. Depending on the application needs, the performance of the immobilized laccase was also assessed by a dye decolorization test [111] or by the degradation rates of different phenolic compounds [16]. Similarly, immobilized peroxidase on Fe_3_O_4_-decorated polyacrylonitrile (PAN) nanofibers was evaluated for its phenol removal efficiency in the presence of hydrogen peroxide over five cycles for performance and reusability [183]. Enzyme activities in both the forward and reverse directions were employed in a case where carbonic anhydrase, which catalyzes the interconversion between CO_2_ and bicarbonate ion, was immobilized in a hydrogel, filling the inter-fiber spaces of a hollow fiber membrane reactor. This configuration enhanced the selective separation of CO_2_ from a higher CO_2_ concentration feed gas, flowing through one set of hollow fiber lumens, to a lower concentration sweep gas, flowing through adjacent hollow fibers [158,159]. In a related application, the performance of immobilized carbonic anhydrase was evaluated only for the forward rate of CO_2_ absorption from gas into water as bicarbonate [155], while for an artificial lung application, the reverse desorption reaction was evaluated, where CO_2_ as bicarbonate ions in the liquid (blood) was converted into CO_2_ gas molecules, carried away by air flow [12,13].

### 7.3. Enzyme–Support Interactions and Structural Changes

When enzymes are immobilized, their conformations, overall shapes, exposure to chemical substrates, and stability against external stresses can all change compared to their free enzyme form, due to their interactions with the support. Techniques that enable observation of these structural changes play critical roles in discovering the origins of emerging or decaying properties of immobilized enzymes. Substantial analytical difficulties arise when enzymes are attached to solid surfaces. Below, the most commonly used techniques are reviewed, pointing out techniques that work well or could be adapted to enzymes on fibrous supports, and emphasizing issues that prevent the widespread use of certain methods as a foundation for new method development and extending their applications.

Circular dichroism (CD) spectroscopy is the “standard” for characterizing secondary structures for “native” proteins dissolved in solutions, and to some extent this method can be used to detect secondary structural changes when enzymes are adsorbed on nanoparticles [128] or undergo chemical modifications [52]. Generally, challenges associated with using CD spectroscopy for immobilized enzyme structural characterization come from interference by the solid support materials. A special case of utilizing CD spectroscopy for immobilized enzymes on fibrous supports involves carbonic anhydrase affinity binding on SWCNTs, where homogenous stable suspensions of the samples were prepared [261]. Another method involved depositing enzyme samples on multiple transparent thin quartz cover slips, that were then stacked together to enhance the signal coming from the enzymes [96]. This technique could potentially be applied by depositing thin electrospun fibers containing enzymes directly onto transparent quartz surfaces, which would solve both agglomeration and sedimentation issues.

While CD spectroscopy provides only a global characterization of the secondary structures of the enzyme, nuclear magnetic resonance (NMR) can provide information on molecular interactions at atomic resolution. Regular solution ^1^H NMR was used for enzymes entrapped in a silica gel network made using deuterated precursors, which are effectively invisible to the proton resonance probe [262]. Enzymes that rotate freely inside the cavity of the gel network experience rapid averaging of the orientation-dependent interactions and generate good peak resolution. Peak broadening is thus associated with slower molecular re-orientation motions caused by restrictive interactions, such as ionic attractive forces of oppositely charged enzyme and gel cavity walls. Unlike deuterated solvents, which are readily available commercially, deuterated support materials are not commonly available or easy to make, which prevents the wide application of this method. Another way of increasing the enzyme-to-support signal ratio is to label the enzyme with some of the less naturally populated nuclei, such as ^15^N, by using ^15^N isotope-enriched growth media to produce the enzyme. Then, ^15^N signals, mostly originating from unbound enzymes, can be followed over time after being brought in contact with support materials to sense the interaction between the enzyme and the support [128]. In solid-state NMR, the signals from less abundant nuclei, such as ^13^C, ^15^N, and ^29^Si, can be significantly enhanced, and their peak broadening can be effectively canceled out using cross-polarization magic angle spinning techniques (CP/MAS) [263]. Nevertheless, high capital and operational costs of high-field, solid-state NMR have hampered its adoption. With progress in producing deuterated enzyme-entrapping polymeric materials, such as deuterated chitosan from filamentous fungus and yeast [264], and decreases in instrumentation costs, more applications of NMR in characterizing immobilized enzymes will emerge.

Small-angle neutron scattering (SANS) is another suitable technique for characterizing enzymes in solution and is extremely sensitive at a length scale that corresponds to the overall shapes of enzymes. For example, SANS exhibited high precision in obtaining the shapes of a series of partially unfolded enzymes [45]. Contrast matching, which varies the ratio of hydrogen to deuterium in the solvent to match out a specific component with the same scattering length density (SLD) and only looks at the remaining parts, can be used to “match out” a gel matrix, making it possible to accurately determine the oligomerization states of proteins entrapped in the pores of a gel [265]. In another example, Jung et al. used perfluoropetane (C_5_F_12_) to match out a silica mesocellular foam. When the pores were loaded with enzyme, effectively excluding C_5_F_12_ from that same space, the scattering from the enzyme in the pores was used to prove the location and size of the enzyme aggregate [266]. The main drawback, unrelated to the technical features of the technique, is the limited accessibility to neutron facilities for most researchers.

Fourier-transform infrared spectroscopy-attenuated total reflection (FTIR-ATR), which is widely available to most researchers, is useful as a simple surface-sensitive technique for monitoring the change in the secondary structure of proteins without additional preparation. The energy associated with the carbonyl bond stretching in the protein backbone, commonly assigned as the amide I band, is highly sensitive to its surrounding chemical environment and will therefore generate well-resolved peaks, corresponding to the α-helical and β-sheet secondary structures [96]. The relative intensities of deconvoluted carbonyl peaks can be used to monitor enzyme structural changes upon immobilization.

A more elaborate but powerful label-enabled method, namely fluorescence resonance energy transfer (FRET), was used to follow enzyme denaturation and renaturation cycles on a support surface [267]. FRET utilizes the distance dependence of the efficiency (inversely proportional to the sixth power of the dye-to-dye distance) of the transfer of energy between a pair of light-sensitive dye molecules. The locations of donor and acceptor dye molecules are chosen so that an efficient energy transfer can occur in the native conformation, whereas it is significantly hindered in the denatured state due to the larger distance.

In some cases, signals that can be used to monitor the denaturation of the enzyme originate from the native structure of the enzyme itself. For example, the absorbance of the heme group in horseradish peroxidase (HRP) at 402 nm can be followed for folding–unfolding equilibrium measurements in solution [52]. The same method should be applicable to immobilized enzymes, provided that the support material can be made sufficiently transparent to visible light.

### 7.4. Enzyme Distribution and Orientation

Information regarding enzyme distribution can be obtained through various imaging and mapping techniques. For example, fluorescence microscopy was used to reveal precise spatial control of a reactive polymer coating through a pattern-masking technique during photo-polymerization and the precise attachment of enzymes to the targeted areas [211]. It was also used to observe the distribution of fluorescein isothiocyanate (FITC)-labeled enzymes in a frozen hydrogel slice [159]. In another study, fluorescein-labeled protein was used as a model to contrast the different immobilization qualities of the untreated and surface-activated polystyrene (PS) electrospun fiber mats, where the activated surface showed clear fiber morphology under fluorescent mode, while the untreated sample was completely dark, confirming the enzyme attachment on activated fiber surfaces [229]. When operated in fluorescent mode, confocal laser scanning microscopy (CLSM) is able to scan transparent samples layer-by-layer and detect labeled enzyme distribution within a solid, as well as a labeled substrate and a fluorescent product [150]. A depth profile of the intensity of the signals provides information on the distribution of the enzyme relative to the surface. In another example using CLSM, the fluorescent images of bovine serum albumin-fluorescein isothiocyanate (BSA-FITC) immobilized on nylon-6,6 electrospun fibrous membranes using either adsorption or covalent attachment were compared [246]. The brightness provided information regarding the amount of immobilized enzyme, and the evenness of the color demonstrated the even distribution of the enzyme.

**Figure 6:**
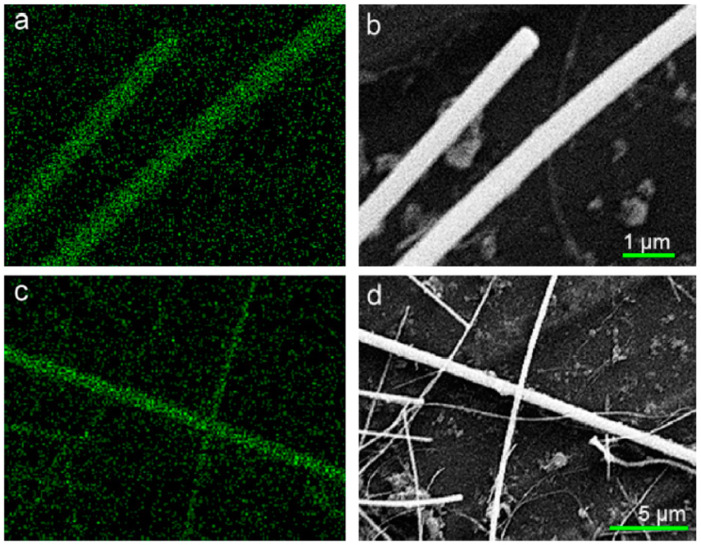
SEM-EDX calcium mapping (**a**,**c**) of glutaraldehyde crosslinked electrospun cellulase enzyme microfibers shown in (**b**,**d**) respectively. Green color in (**a**,**c**) corresponds to Ca^2+^ complexed at Ca^2+^-binding domains in cellulase. (reprinted with permission from [257], Copyright 2011, Elsevier).

In an early study, Solas et al. demonstrated the detection of elemental iron coming from catalase adsorbed on the surface of a fibrous bioskin, which was a copolymer of glucosamine and N-acetyl galactosamine, using an energy-dispersive X-ray spectroscopy (EDX) technique [248]. The good lateral resolution of EDX allowed Tran et al. to confirm the formation of glutaraldehyde crosslinked cellulase enzyme “microfibers”, that were fabricated by concentric electrospinning with a sacrificial PEO shell, by detecting Ca^2+^ ions that were complexed in the enzyme’s binding domains [257] (Figure 6). Han et al. demonstrated the mapping of sulfur, a non-metal element, contained in the enzyme to evaluate enzyme distribution on a core-sheath electrospun nanofiber mat, with the enzyme comprising the sheath [174].

Additionally, being a surface-sensitive elemental analysis technique that has mapping capability, X-ray photoelectron spectroscopy (XPS) can be used to map protein distribution through elemental mapping. For example, the changes in the nitrogen atomic percentage from XPS data were used to monitor the protein coverage, i.e., distribution, on surfaces [268]. However, due to its coarse lateral resolution (>3 μm), simultaneous observation of the finer fiber morphology through elemental mapping has not been reported. Another surface characterization method having the ability to extract chemical bonding and elemental information from the top few layers of the surface is time-of-flight secondary ion mass spectrometry (TOF-SIMS). Tyler et al. demonstrated the use of triatomic Bi_3_^+^ as the primary ion and a maximum autocorrelation factors (MAF) image-processing method for generating a clear contrast (boundaries confirmed by fluorescence microscopy) between two similar proteins (human serum albumin versus bovine serum albumin) and two very different proteins (human serum albumin versus hemoglobin) immobilized on non-flat polystyrene micro-bead surfaces [269]. This demonstration of the technique on complex-shaped surfaces instead of regular flat substrates, such as silicon wafers, indicates that TOF-SIMS can also be applied to study the distribution of enzymes immobilized on fibrous supports, as was carried out with protease incorporated in poly(ethyleneoxide) solution, blown, nonwoven webs [270]. The technical parameters of different surface characterization techniques are compared in Table 7.

In some limited cases, enzyme layers can be directly observed on extremely thin fibers. Tavares et al. observed an adsorbed laccase layer on MWCNTs at a <10 nm scale using transmission electron microscopy (TEM) [247]. Mohamad et al. observed an increase in the diameter of the acid-functionalized MWCNT after lipase adsorption using a TEM technique [253]. Chen et al. used both TEM and atomic force microscopy (AFM) for estimating enzyme layer thickness and distribution [261]. They showed that carbonic anhydrases modified with CNT-binding peptides were immobilized onto SWCNTs through affinity binding as a single layer corresponding to the diameter of the enzyme. In addition, individual enzyme particles were discernable by AFM based on differences in enzyme and SWCNT hardness.

**Figure 7:**
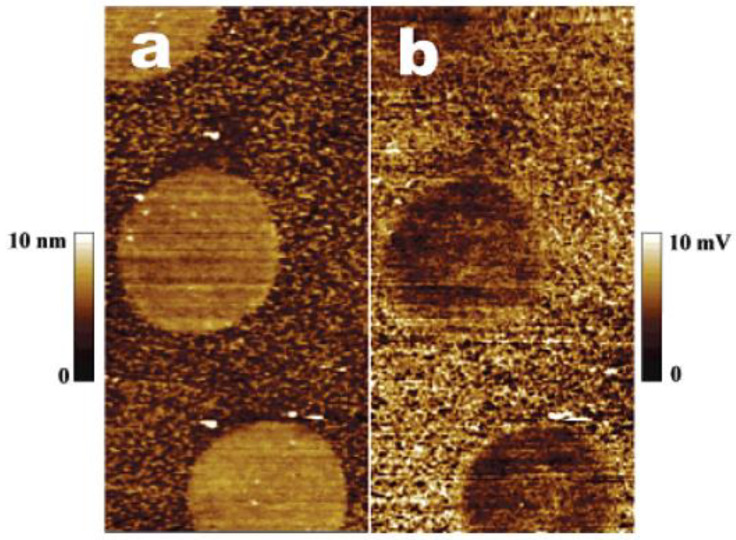
AFM topographic (**a**) and frictional (**b**) images of the CA-immobilized surface (image size: 4.5 µm × 9 µm). The dot was functionalized with negatively charged thiol 16-mercaptohexadecanoic acid and the remaining unstamped region was functionalized with positively charged thiol 6-mercaptohexyl-N-pyridinium bromide before immobilization (reprinted with permission from [271], Copyright 2006, American Chemical Society).

When modified with a suitable ligand, AFM probes are able to gauge specific ligand–protein interactions. Wang et al. attached thiolated sulfonamide, a carbonic anhydrase (CA) inhibitor, on an AFM probe tip and monitored unbinding forces between the tip and the CA immobilized by electrostatic interactions on two oppositely charged surfaces [271]. Due to an overall positive charge near the opening of the active site, CAs immobilized on a negatively charged surface are expected to have their active site facing toward the surface. Conversely, CAs immobilized on a positively charged surface will have exposed active sites. The orientation was confirmed by the different unbinding forces corresponding to a strong active site–inhibitor interaction and a weak non-specific interaction, the contrast of which clearly portrays the boundary of the underlying microarray pattern (Figure 7).

TOF-SIMS was also capable of probing the orientation of immobilized enzymes. It may be apparent from its short detection depth of only 1–2 nm and a typical enzyme diameter of >3 nm that only the side of the enzyme that is exposed to the primary ion beam will be detected. By this method, the surface amino acid profile [272], or simply the ratio of asymmetrically located amino acids peaks [268], was used to deduce the immobilized protein orientation on a silicon wafer surface. However, studies extending the application of TOF-SIMS into probing immobilized enzyme orientation on flexible fibrous materials are still lacking, for obvious reasons, such as the complex geometry and flexibility. With the growing interest and impetus in developing biocatalytic fibrous materials, method development efforts for solving this problem will likely follow.

## 8. Concluding Remarks and Future Direction

Combinations of multiple support materials (e.g., at different geometric scales) are a growing trend in fabricating biocatalytic materials, especially where fibrous membranes are the major physical support in the integrated structure. With these combinations, advantages of one type of support can be integrated into another, resulting in hybrid catalytic membranes that have characteristics of a high surface area, chemical functionality on the surface, the capability of stabilizing enzymes, and ease in recycling immobilized biocatalysts. These characteristics address the overarching goals of enzyme immobilization, which are to promote enzyme longevity, thereby reducing costs, and to provide reactive versatility through the diverse catalytic function of enzymes.

Extended longevity is a critical requirement for commercial adoption of biocatalytic materials in continuous processes; however, durability experiments are non-trivial with respect to time and cost. Consequently, most research still primarily focuses on developing new approaches for immobilization and broadening enzyme activity to accommodate application conditions (substrate selectivity, pH, temperature, etc.), rather than extending longevity. Even for these studies, multiple different instrumental characterizations are necessary to thoroughly understand enzyme immobilization mechanisms, enzyme-immobilized materials, and the processes they catalyze. The lack of systematic characterization of these complex systems is one reason why enzyme immobilization studies are largely presented on a case-by-case basis. Fortunately, the toolbox and knowledge sharing of quantitative characterization methods is increasing. This will lead to improved characterization of biocatalytic materials in general and will guide the future development and scale-up of heterogeneous biocatalytic membranes, including those with advantageous hierarchical fibrous structures, for industrial applications.

## Figures and Tables

**Table 1:** Enzyme classes of the NC-IUBMB enzyme list [20].

Class	Name	Catalyzed Reaction
1	Oxidoreductases	AH2 + B = A + BH2 or AH2 + B+ = A + BH + H+
2	Transferases	AX + B = A + BX
3	Hydrolases	A-B + H_2_O = AH + BOH
4	Lyases	A = ^1^ B + X-Y = X-A-B-Y
5	Isomerases	A = B
6	Ligases	A + B + NTP = A-B + NDP + P or A + B + NTP = A-B + NMP + PP

^1^ Refers to the double-bond between A and B.

**Table 2:** Metrics for quantifying (immobilized) enzyme performance.

Metric	Reference
$Enzyme\;Activity\left({U\;}^{†}\right)=\frac{Amount\;Substrate\;Converted\;(\mu mol)}{time\;(min)}$	Common knowledge
$Specific\;Enzyme\;Activity\;(U\;{mg}^{-1})=\frac{U}{Amount\;Enzyme\;Protein\;(mg)}$	Common knowledge
$Immobilization\;Yield\left(\%\right)=\frac{Quantity\;of\;Enzyme\;Immobilized}{Total\;Amount\;of\;Enzyme\;Used}\times 100\%$	[49]
$Activity\;Recovery\left(\%\right)=\frac{Immobilized\;Enzyme\;Activity}{Free\;Enzyme\;Activity}\times 100\%$	[49]
$Enzyme\;Loading\left(\%\right)=\frac{Mass\;of\;Enzyme\;Immobilized}{Total\;Mass\;of\;Immobilized\;Material}\times 100\%$	[58]
$Catalyst\;Consumption\left(g\;{kg}^{-1}\right)=\frac{Amount\;Enzyme\;(g)}{Amount\;Product\;(kg)}$	[59]
${STY\;}^{‡}\left(g\;L^{-1}\;h^{-1}\right)=\frac{Amount\;Product\;(g)}{Reactor\;Volume\left(L\right)\times Unit\;time\;(h)}$	[59]
$Productivity\left(kg\;g^{-1}\right)=\frac{Amount\;Product\;(kg)}{Amount\;Enzyme\left(g\right)\times Unit\;time\;(h)}\times Longevity\;(h)$	Inspired by [60]

^†^ U: enzyme activity unit; ^‡^ STY: space–time yield.

**Table 3:** Examples that quantify fibrous membrane immobilized enzyme performance.

Enzyme	Immobilization Approach	Metrics and Outcomes	Reference
*Pseudomonas* *cepaciae* lipase	Physically adsorbed enzymes on carbon fiber or glass fiber woven fabrics	Enzyme loading 1–20 mg/g fiber. Immobilization yield > 90%. No loss of activity at 30 or 45 °C and 80% activity retention at 60 °C after 33 h of continuous reaction in gas phase hydrolysis of ethyl acetate. Stated that 5 sequential runs were achieved with minimal activity loss.	[169]
*Candida* *antarctica* Lipase B	Covalently immobilized onto polymeric electrospun membrane	Enzyme loading 25–48 mg/g fiber. Specific activity 1551–2555 U/mg. Retained 50% of initial activity after 15 cycles, over 65% after 10 h of heat incubation, and over 75% after 30 days of storage.	[187]
*Candida* *antarctica* lipase B	Covalently immobilized onto polymeric electrospun membrane	Enzyme loading 30–135 mg/g fiber. Specific activity 0.3–0.9 U/mg protein. Activity retention (thermal stability) 43% for free lipase and 79% for immobilized lipase at 40 °C for 3 h. 62% activity after 7 reuses and nearly 75% after being treated in methanol for 12 h at 35 °C.	[153]
*Candida rugosa* lipase	Covalently immobilized onto collagen fibers containing magnetic particles	Immobilized lipase reached 2390 U/g under optimal conditions. 5 times longer storage stability at 4 °C compared to free enzymes.	[166]
*Candida rugosa* lipase	Covalently immobilized onto regenerated cellulosic electrospun fibers	Retained up to 80% activity when exposed to organic solvents. 100% retained activity after one catalytic cycle (7 h).	[113]
Lysozyme	Covalently immobilized onto activated wool fabrics	Optimal protein recovery was 48%. Retained 43% of its catalytic activity after five cycles of use.	[188]
Trypsin	Covalently immobilized onto woven PLA	Optimized specific activity ~3.8 U/mg. Retained > 55% of initial activity after 20 days of storage and demonstrated activity after 15 cycles.	[189]
Bovine carbonic anhydrase	Covalently conjugated enzyme adsorbed onto glass fiber surface	Enzyme conjugates immobilized on glass fibers have 3–4 times catalytic activity (~14.7 × 10^−4^ U/cm^2^), compared to those immobilized on smooth glass surface. Enzyme conjugates immobilized on glass fibers have around 2 times catalytic activity compared to immobilized free enzymes.	[190]
*Thermomyces lanuginosus* lipase	Layer-by-layer self-assembly on cotton cloth	The best catalytic activity found with four enzyme layers (13 U/cm^2^).	[191]
α-chymotrypsin	Covalently immobilized onto polystyrene and co-polymer electrospun nanofibers with/without the presence of glutaraldehyde	Immobilized enzymes showed a 3–7 times longer half-life compared to free enzymes. Aggregated immobilized enzymes showed 9 times activity compared to a single layer.	[192]
Yeast alcohol dehydrogenase	Covalently immobilized onto modified polyvinyl alcohol-knitted fabrics with glutaraldehyde as the spacer	Immobilized enzymes retained 46% and 27% activity at pH 9 and 10.5, respectively, at which free enzymes lost all their activity. Retained > 60% activity at high temperature where free enzymes are inactivated. Fabrics showed > 60% activity after 8 catalytic cycles.	[193]
Catalase	Covalently immobilized to PET and nylon fabrics by photochemical treatment	Enzyme loadings reach 20–33 mg/g when surfactants are used. 10% retained activity compared to free catalase. Retained enzyme activity after 20 catalytic cycles.	[194]
Glucose oxidase	Entrapped in silk fibroin gel then applied to non-woven fabrics	Activity recovery from 5–94%, depending on enzyme concentration. Apparent activity ranged from 6–112 U/mg compared to 120 U/mg of free enzyme.	[10]
*Candida rugosa* lipase	Entrapped in water-soluble electrospun fibers followed by crosslinking	Enzyme loading expressed as 50% enzyme-to-polymer ratio (wt.%). 85% activity retained after 40 °C storage for 4 h, at which free lipase lost almost 70% activity in 1 h.	[151]
Vairous microbial lipases	Entrapped in electrospun poly(vinyl alcohol) (PVA) nanofibers	Entrapped enzymes have 1.5–90 times activity recovery compared to free enzymes, depending on the agents used in the entrapment. Retained activity was reported after at least 8 catalytic cycles.	[172]
*α-*amylase	Entrapped in electrospun poly(vinyl alcohol) (PVA) nanofibers	38–52 mg·g^−1^ enzyme loading, depending on the concentration. Reported specific activity of 0.3–0.45 U·mL^−1^ ·mg^−1^ of immobilized enzyme. About 2–3 times retained activity compared to free enzymes at 80 °C for 10 min.	[195]
*α-*amylase	Entrapped in ethyl cellulose electrospun fibers	2-fold increase in storage stability compared to free enzyme. Membrane retained 100% and 50% after 10 and 15 catalytic cycles.	[196]
Laccase	Entrapped in γ-cyclodextrin then electrospun into poly(ε-caprolactone) (PCL) fibers	11–96 U/mg depending on the preparation method.	[197]
Hyper-thermophilic α-galactosidase and β-glucosidase	Entrapped in PVA electrospun nanofibers through the presence of HCl as the cross-linking initiator	2-fold improvement in enzyme thermal stability (90 °C and pH 5.5).	[170]

**Table 4:** Common physical parameters for fibrous supports.

Parameters	Methods	Units
Fabric weight	Area and mass	g/m^2^
Yarn linear density	Length and mass	Tex (g/km) Denier (g/9000 m)
Yarns per inch	Length and counting	numbers of yarns/inch in each direction
Fabric thickness	Length under pressure	mm
Fiber diameter	Length from microscope (optical or electron)	µm or nm
Surface area	Nitrogen adsorption-desorption	m^2^/g
Pore volume	Nitrogen adsorption-desorption	cm^3^/g
Pore size	Nitrogen adsorption-desorption, capillary flow porometer (through pores)	µm or nm

**Table 5:** Specific surface area examples for fibrous supports (see Supplementary Materials Table S1 for additional information about these studies).

Material	Physical Structure	Specific Surface Area (m^2^ g^−1^)	Ref.
Glass or carbon fibers	Woven fabrics	2	[169]
Multi-walled carbon nanotubes (MWCNTs)	MWCNTs with 10–20 nm diameters and 5–15 μm length	73	[247]
Cellulose acetate microfibers	Electrospun non-porous and porous fibers, with or without montmorillonite	1.94–11.87	[244]
Polyaniline nanofibers	Nanofibers	58.4	[243]
Regenerated cellulose	Nanofiber membrane	5.3	[113]
Poly(acrylic acid)-coated polypropylene fibers	Non-woven fabric	0.395	[245]
Collagen composite fibers	Porous collagen composite fibers with magnetic Fe_3_O_4_ particles	11.59	[166]
Cellulose fibers	Commercial filter paper	~1	[211]

**Table 6:** Techniques for direct enzyme loading measurement on support materials.

Technique	Sample Requirement	Detection Limit	Principle
AAS	Metalloenzymes	ppb	Absorption of lights at characteristic wavelengths of the free metal ions
ICP-OES (ICP-AES)	Metalloenzymes	ppb	Emission of lights at characteristic wavelengths of the excited atoms and ions
TGA	High enzyme loading and thermal stable support	%	Difference in thermal decomposition temperatures of the enzyme and support

**Table 7:** Comparisons of surface characterization methods.

Technique	Information Provided	Depth	Type of Analysis	Detection Limit
TOF-SIMS	Chemical bonding and elemental	~1–2 nm	Mostly qualitative, or semi-quantitative, standard difficult to prepare	0.01–0.1 at % atomic concentration
XPS	Chemical bonding, oxidation state, and elemental	~5 nm	Quantitative	0.1–1.0 at % atomic concentration
EDX	Elemental	1–2 µm	Mostly qualitative, or semi-quantitative, requires standard for quantitative analysis	0.05 wt.%
FTIR-ATR	Chemical bonds, interactions in the solid state	0.5–2 µm depending on wavelength of the light	Mostly qualitative, requires calibration with a second technique for quantitative analysis	0.1 wt.%

## Data Availability

Not applicable.

## Supplementary Materials

The following supporting information can be downloaded at: https://www.mdpi.com/article/10.3390/membranes13050532/s1, Table S1: Summary of techniques for enzyme immobilization on fibrous membranes.
