# Supplementary material for: Developing Enzyme Immobilization with Fibrous Membranes: Longevity and Characterization Considerations
Source: Membranes (Basel). 2023 May 20;13(5):532. doi: 10.3390/membranes13050532 (PMC10221158; doi:10.3390/membranes13050532)
Supplement: Supplementary file 1 [file membranes-13-00532-s001.zip › membranes-2281135-supplementary.pdf]

Review

Developing Enzyme Immobilization with Fibrous Membranes: Longevity and Characterization Considerations

Yue Yuan [1,2], Jialong Shen [2] and Sonja Salmon [2,*]

[1] Center for Nanophase Materials and Sciences, Oak Ridge National Laboratory, Oak Ridge, TN 37830, United States.
[2] Fiber and Polymer Science Program and Department of Textile Engineering Chemistry & Science, North Carolina State University, Raleigh, NC 27695, United States.
* Correspondence: sisalmon@ncsu.edu

Supplementary Materials

Contains 1 Table

Table S1. Summary of Techniques for Enzyme immobilization on Fibrous Membranes

Support material composition	Enzyme/location	Physical structure	Characterization	Ref
**Direct coating**				
Cellulose monoacetate / Chitosan blend with NaOH treatment and optional glutaraldehyde activation.	Protease/ direct coating of enzyme solution on the nano/micro fibrous membrane.	Electrospun nano/micro fibrous membrane.	FTIR for chemical composition, DSC for observing shift in thermal signature of each component of the blend, TGA for change in thermal degradation of the blend from varying the ratio. Activity assay for reusability test.	[1]
**Affinity binding**				
Surface modified cellulose fibers with either carboxylic acid or tertiary amine functionalities were further decorated with biotin through amide bond formation using activated succinate ester.	Ureases decorated with biotins form strong non-covalent bond with biotins attached on cellulose fiber surfaces through avidin-biotin complexation.	Non-woven textile fabrics with specific mass of 450 g/m^2.	Urease activity measurement in an electro-dialysis cell.	[2]
Single-walled carbon nanotube (SWNTs)	Carbonic anhydrase (CA) with carbon-nanotube-binding peptides (CBP) expressed at the C terminal/ affinity binding to SWNTs	SWNTs with an average diameter of 2 nm and length of 1 µm.	TEM and AFM for morphology and estimating enzyme layer thickness and distribution. CD spectroscopy and Raman spectrum for analysis of the interaction between CA and SWNTs. Activity assay for pH profile.	[3]
**Non-covalent adsorption**				
Aluminoborosilicate glass fiber and carbon fiber	Lipase/physical adsorption on the surface of the fibers	Woven fabrics with specific surface area of 2 m^2/g	SEM for morphology. Activity assay to estimate enzyme loading. Gas chromatography to quantify substrate and product in the laboratory scale solid–gas bioreactor.	[4]

Polysulfone containing PVP or PEG	Lipase/physical adsorption onto fiber surfaces or into pores.	Electrospun nanofibrous membrane	SEM for morphology and fiber diameter. Bradford protein assay for enzyme loading. Activity assay for pH and temperature profiles, thermal stability, and kinetic parameters.	[5]
Polyaniline	L-asparaginase/ adsorption on nanofibers and in the porous structures	Polyaniline nanofiber-formed *in situ* during polymerization	XRD, XPS, FTIR for surface chemistry. TEM and SEM for morphology. Enzyme activity assay for pH and temperature profiles and enzyme kinetics.	[6]
Multi-walled carbon nanotubes (MWCNTs)	Laccase/ adsorption on MWCNTs	Multi-walled carbon nanotubes (MWCNTs) with diameters of 10–20 nm, length of 5–15 μm and surface area of 73 m^2/g	SEM for fibrous morphology, TEM for observing adsorbed enzyme layer (<10 nm scale), FTIR and Raman for the interaction between the enzyme and MWCNTs. Activity assay for the effects of contact time, pH, and enzyme concentration for adsorption, kinetic parameters, thermodynamic parameters, reusability, and storage stability.	[7]
Cellulose acetate w/ or w/o montmorillonite	Laccase/ Adsorption on the non-porous fiber surfaces or porous fiber surfaces and pores, or on montmorillonite surfaces	Electrospun non-porous (~740 nm) and porous (~3.21 μm) fibers w/o montmorillonite doping. BET surface area ranges 1.94-11.87 m^2/g.	SEM and TEM for morphology, porous structures. N_2 adsorption–desorption isotherms for surface area and pore size. Activity assay for temperature and pH profiles, storage stability, and reusability.	[8]
Borosilicate glass/ Poly(methacrylate)-based dendronized polymer (denpol)	Carbonic anhydrase / Enzymes were attached to denpols via bis-aryl hydrazone bond before non-covalent adsorption of denpol-enzyme conjugate on glass fiber surfaces.	Commercial binder-free glass microfiber filter	SEM for morphology. Spectrophotometer for quantifying conjugation yield, for monitoring product concentrations to evaluate reusability, and for thermal stability.	[9]
**Non-covalent ionic or electrostatic adsorption**				
Bioskin: Copolymer of glucosamine and N-acetyl galactosamine	Catalase/Adsorbed on the fiber surface through ionic interaction	Commercial bioskin sheet composed of fibers	Lowry protein assay. Apparent Michaelis Constant. Room temperature storage stability. SEM with EDS.	[10]
Cellulose	Lipase/Alternating ionic adsorption of PEI and enzyme or hydrophobic aggregation of enzyme alone, both layer-by-layer.	Cotton flannel cloth, 15 mg/cm^2	Activity assay for accessing and comparing the enzyme deposition using the two different adsorption strategies.	[11]

Wool	Catalase/electrostatic layer by layer deposition of alternating positively charged poly (diallyldimethylammonium chloride) layer and negatively charged catalase layer on wool fiber surface.	Commercial wool fabric (135 g/m2)	SEM for fiber morphology. Bradford protein assay for enzyme loading. Zeta potential and relative color depth resulting from dyeing with cationic dye demonstrated the alternating surface charge. Activity assay for temperature and pH profile and reusability.	[12]
Polypropylene	Carbonic Anhydrase / Layer by layer adsorption between polyelectrolyte, such as PEI, PSS, and PAH, and enzymes with/without the addition of mesoporous silica to increase enzyme loading.	Flat sheet PP membrane with a pore size of 0.10 μm and a thickness of 75−110 μm. Additionally, mesoporous silica layer was coated to increase enzyme loading.	SEM for morphology. Zeta-potential to characterize charge alternation during layer-by-layer deposition. Quartz Crystal Microgravimetry for estimating the mass of polyelectrolyte or enzyme deposited on the surface.	[13]
Multi-walled carbon nanotubes (MWCNTs) treated with HNO3 and H_2SO_4.	Lipase/ adsorption on acid functionalized MWCNT surfaces	MWCNT	SEM and TEM for morphology and evidence of enzyme layer. TGA and protein content for approximate enzyme loading. FTIR for confirming acid functionality and enzyme adsorption. Activity assay for thermal stability. NMR and Gas Chromatography for confirming the product chemical structure.	[14]
Cellulose with adsorbed carboxyl methyl cellulose assisted chemical binding of silver ion for subsequent *in situ* reductive formation of silver nanoparticle.	Laccase/ adsorption through electrostatic interaction with silver nanoparticle	Cellulose nanofiber made by deacetylation of cellulose acetate nanofiber, diameter of 370 ± 174 nm	SEM and TEM for morphology. EDS for identifying silver nanoparticle. FTIR for identifying components of the composite. Electrochemical tests for application properties.	[15]
Polypropylene (PP) or Polydimethoxysilane (PDMS)	Carbonic anhydrase/ electrostatic layer by layer adsorption assisted by alternating polyelectrolytes	PP hollow fiber porous membrane with pore diameter of 0.2 μm and porosity of 0.5, or PMDS nonporous hollow fiber membrane.	SEM for morphology, pore size etc. Hollow fiber membrane contactor for mass transfer coefficient.	[16]
Cellulose	Pectinase / layer by layer (LBL) adsorption by alternately disperse pulp fibers in PEI solution	Bleached kraft softwood pulp	Nitrogen content for calculating PEI and enzyme loading. Zeta-potential for monitoring alternating surface charge in the LBL process. Activity assay for determining the number of	[17]

	and pectinase solution with filtration and DI water rinsing after each adsorption layer.		layers reaching diffusion limit, pH and temperature profiles, reusability, and kinetic parameters.	
**Non-covalent adsorption then crosslinking**				
Polyaniline	Glucose oxidase/adsorption, precipitation using ammonium sulfate, and crosslinking using glutaraldehyde performed in sequence on polyaniline nanofibers.	Polyaniline nanofiber formed *in situ* during polymerization. BET surface area 58.4 m^2/g with average pore diameter of 12.26 nm and pore volume of 0.179 cm^3/g	SEM for morphology. Activity assay for enzyme stability. Measured power density and electrochemical impedance spectra of the enzyme anode for application performance and stability. Electrochemical impedance spectra for charge.	[18]
Polylactic acid doped with graphene oxide or nano-sized metal organic framework	Carbonic anhydrase/adsorption on fiber surfaces with or without glutaraldehyde crosslinking.	PLA or PLA GO/n-MOF composite electrospun membrane with fiber diameter of ~4.38 ± 1.5 μm	SEM for morphology. XRD for crystal structure. Tensile test for the mechanical property of the support membrane. Activity assay for temperature and pH profile, kinetic parameters, reusability, and storage stability. Confocal laser scanning microscopy for visualizing enzyme distribution.	[19]
Poly (vinyl alcohol) (PVA) or Poly (ethylene oxide) (PEO) with Sodium alginate	Lipase/ first adsorption on fiber surfaces and then crosslinked by glutaraldehyde (GA)	Electrospun composite nanofibers with diameters of 150- 370 nm Crosslinked chemically with GA and ionically with Ca^{2+}	FTIR for confirming chemical composition. TGA for thermal stability. SEM for morphology. Activity assay for thermal stability, pH profile, reusability, and kinetic parameters.	[20]
**Covalent surface attachment**				
Surface modified Nylon 6,6. React the free carboxyl groups with carbodiimide and diamine forming additional amino groups on the surface	Tyrosinase/surface covalent attachment by glutaraldehyde crosslinking	Membrane disk with pore size of 0.2 or 10 μm	Kinetic parameters by fitting modified Michaelis-Menten model accounting for inactivation and product inhibition for the study of the effects of oxygen partial pressure and scaling-up. Enzyme stability by repeated batch experiments.	[21,22]
Glycine esterified cotton	Lysozyme/surface amide bonds formed between amino groups on glycine esterified cotton and aspartate or glutamate residues on the lysozyme.	Desized, scoured, bleached, and mercerized cotton twill.	Kjeldahl method for enzyme loading. Antibacterial assay based on lysis of cells (drops in O.D.) for end-use performance.	[23]

Polystyrene containing 4-nitrophenyl formate ending groups which is reactive toward amine groups in enzyme	α-chymotrypsin covalently bonded to polystyrene chain ends through urethane linkages.	Electrospun nanofibers with diameters ranging from 120 nm to 1 μm.	SEM for fiber morphology and diameter measurement. Enzyme hydrolytic activity in both aqueous and non-aqueous solution. Enzyme stability in Methanol.	[24]
Scoured and bleached cotton fabric activated with cyanuric chloride as anchor molecules	Catalase covalently bonded to anchor molecules on the cotton fiber surfaces forming monolayer. Additional layers were attached using glutaraldehyde crosslinking.	Cotton fabric, 102g/m^{-2}	Atomic absorption spectroscopy (ASS) for enzyme loading. SEM for surface morphology.	[25]
Regenerated cellulose nanofibers made by deacetylation of the cellulose acetate nanofibers	Lipase/ amino group on enzyme covalently bonded to carboxyl group of the PEG spacer through amide bonds formation facilitated by carbodiimide (EDC) activation	Nanofiber membrane with fiber diameter of 500 nm and specific surface area of 5.3m^2/g	FTIR, DSC, TGA confirmed the deacetylation and esterification. Titration for free acid amount. SEM for morphology. Activity assay for evaluation of temperature and pH profiles, solvent stability, reusability and immobilization conditions.	[26]
Poly(styrene) and poly(styrene-co-maleic anhydride) blend	α-chymotrypsin/first monolayer of enzymes form amide bonds with anhydride groups on the fiber surface. Additional layers of enzymes bonded onto the prior layers using GA as cross-linker forming 3-D crosslinked enzyme aggregate on fiber surface.	Electrospun nanofibers with diameter of ~500 nm	SEM for morphology. Activity assay for enzyme stability after incubation under shaking condition. Reflection–absorption infrared spectroscopy (RAIRS) for functional group identify cation in the polymer blend. Protein assay for detection of enzyme leaching.	[27]
Polypropylene grafted with poly (acrylic acid)	Urease/Covalent attachment on the poly (acrylic acid) coated fiber surfaces through amidation mediated by carbodiimide activation.	Non-woven fabric with starting specific surface area of 0.395 m^2/g	SEM for morphology and fiber diameter used to calculate specific surface area. FTIR for detection of grafted carboxyl functionality. Dimension and weight for grafting mass ratio, swelling ratio, and void fraction. Permeability was measured with DI water. Bradford assay for protein loading. Nessler method and total organic carbon analyzer for measuring urea content. Activity assay for pH and temperature profiles, storage stability and kinetic parameters.	[28]
Poly (acrylonitrile-co-maleic acid)	Lipase/amide bond formed between amino group on enzyme and activated ester on fiber surface using EDC/NHS. Or first tether chitosan or gelatin through amide bond using	Electrospun nanofibrous membrane with fiber diameter of 100 nm.	SEM for morphology and fiber diameter. Bradford protein assay for enzyme loading. Activity assay for pH and thermal stability, reusability, and kinetic parameters.	[29]

	the EDC/NHS coupling agent, then activate surface with GA and then bond enzyme.			
Nylon 6,6 treated with protease which promotes chain scission and create more free amine chain ends	Laccase/Multi-step covalent attachments following the sequence: Free amino chain ends on fiber surface react with GA, GA react with diamine (act as spacer), diamine react with GA, GA react with amino group on enzyme.	Commercial Nylon 6,6 fabric, plain woven structure with 63g/m^2,	Factorial experiment design for the effects of immobilization pH, enzyme dosing, cross-linker concentration, and the use of spacer on the half-life time, protein retention, and immobilization yield.	[30]
Cotton was crosslinked by 1,2,3,4-Butanetetracarboxylic acid forming ester in the presence of alkaline catalyst or coated with PEI crosslinked by GA and additionally chelated by copper	α-amylase, alkaline pectinase, or laccase/ amide bond formation by carbodiimide activation of carboxyl on the ester cross-linked cotton surfaces or physical adsorption onto the copper chelated GA crosslinked PEI on cotton surfaces	Mill-scoured and bleached cotton fabric of 125 g/m^2	Kjeldahl method for nitrogen content post PEI coating. Atomic absorption spectroscopy for copper content. Activity assay and protein assay for activity retention and enzyme loading under different immobilization conditions. Antimicrobial test for application performance when new or its durability after multiple laundry cycles.	[31]
Poly(styrene) and poly (styrene-co-maleic anhydride) blend pre-wetted in alcohol water mixture to help with dispersion in aqueous solution	Lipase/ monolayer of enzymes form amide bonds with anhydride groups on the fiber surface.	Electrospun nanofibers with diameter of ~600 nm	SEM for morphology and fiber diameter. FTIR for functional groups on fiber. Activity assay in batch reaction mode for knetic parameter. Absorbance at 280 nm for protein concentration and enzyme loading. Continuous flow reactor for steady state conversion and apparent rate constant of the reactor.	[32]
Polyacrylonitrile bubble with HCl in absolute ethanol converting nitrile to imidoester groups	Lipase/amino groups on enzyme react with imidoester groups on fiber forming amidine bond.	Electrospun nanofibrous membrane with fiber diameter of 150-300 nm.	SEM for morphology and fiber diameter. FTIR for the presence of enzyme functional groups as well as enzyme-support covalent bond. Water contact angle for the surface property. Tensile test for mechanical property. Batch reaction activity assay for pH and temperature profile, storage stability and reusability.	[33]
Cotton oxidized by sodium periodate forming reactive aldehyde groups	Catalase/surface covalent attachment through Schiff base.	Desized, scoured, and bleached cotton fabric (14.5tex ×14.5tex)	Coomassie Brilliant Blue G-250 for qualitative confirmation of enzyme fixation. Hydroxylamine hydrochloride titration for aldehyde content. Activity assay for optimum temperature and pH for oxidation, temperature and pH profile, and reusability.	[34]

Wool activated by glutaraldehyde	Lysozyme/covalently bonded to aldehyde group on activated wool fibers.	Worsted wool fabric (gabardine 220 g m^{-2}, 410 ends/ 10 cm × 250 picks/10 cm)	Coomassie Brilliant Blue G-250 for qualitative confirmation of enzyme fixation (Modified Bradford method). Activity assay for optimum immobilization condition. Antibacterial activities for performance and reusability.	[35]
Cotton functionalized by citric acid crosslinking (carboxylic acid surface) or aminosilanization (amine surface)	Lysozyme/ on surface of the functionalized surface through amide bond formation mediated by carbodiimide coupling	Desized, scoured, bleached and mercerized cotton printcloth and nonwoven fabric	Carboxyl content by titration, Amino content monitored by N% in elemental analysis. Antibacterial assay based on lysis of cells (drops in O.D.). Zeta potential for surface charges. FTIR for detention amide bonds	[36]
Poly[acrylonitrile-co-(glycidyl methacrylate)] (PANGMA)	Lipase/directly reacting epoxy group on fiber surface with amino group from enzyme or first adding amine spacers and then covalent immobilize enzyme using GA	Electrospun PANGMA nanofiber mat with diameter of 200-300 nm.	SEM for fiber morphology. FTIR for covalent bond formation and enzyme presence. Activity assay (p-NPA) for optimum immobilization pH, thermal stability, storage stability, reusability. Protein assay (Bradford method) for enzyme loading.	[37]
Polymethylpentene (PMP) treated by plasma to create hydroxyl groups which is in turn activated by cyanogen bromide	Carbonic Anhydrase/ cyanate esters or imidocarbonates on activated hollow fiber surface covalently bonded to amino groups on enzymes	Commercial polymethylpentene (PMP) hollow fibers with OD of 380 nm and ID of 200 nm	Esterase activity assay. Application activity assay in oxygenator. Cyanate ester active groups content for optimizing the activation conditions. SEM for hemocompatibility through observing surface deposited platelet amount.	[38]
Nano fibrillated Cellulose functionalized by amine, epoxy, or carboxyl groups	Alkaline phosphatase / Heterobifunctional modification of amino groups conferring aldehyde and hydrazine/hydrazide to enzyme and support, respectively, which then react to form hydrazone bond. Or epoxy amination with the lysine, or EDC/NHS activation of carboxylic acid groups and then form amide bond with amino group on lysine.	Nano fibrillated cellulose, diameter range from 4-100 depending on functionalization method used. Nano fibrils spin coated to form films.	AFM for morphology, MALDI–TOF, CP/MAS NMR, Nitrogen Content for degree of modification. Quartz Crystal Microbalance with Dissipation (QCM-D) for nonspecific adsorption of proteins to surfaces.	[39]
Poly (vinyl alcohol-co-ethylene) (PVA-co-PE) activated by glutaraldehyde (GA)	Lipase/Aldehyde groups on the activated surface react with lysine amino groups on enzyme.	PVA-co-PE nanofibers made by removal of sacrificial cellulose acetate butyrate from their melt extruded	Activity assay for pH and temperature profile, thermal and storage stability, and reusability. SEM for morphology. FTIR for change in chemical structures. Protein assay for enzyme loading. Hydroxylamine hydrochloride titration for aldehyde content.	[40]

		immiscible blend fibers. Diameter of 50-300 nm.		
Chitosan activated by glutaraldehyde (GA)	Lysozyme/Cross-linked and covalently attached to fiber surfaces.	Chitosan nanofiber made by co-electrospinning with and subsequent removing of sacrificial PVA. Fiber diameter smaller than 150-200nm range.	Bradford protein assay for enzyme loading. FE-SEM for fiber morphology. Activity assay for effects of pH and temperature, thermal and storage stabilities, and reusabilities. Antibacterial test for application performance.	[41]
Polypropylene with aminated siloxane coating was decorated with chitosan through glutaraldehyde (GA) activation	Carbonic anhydrase/ Chitosan amino groups on the fiber surface were activated by GA and covalently bonded to amino groups on the enzyme	Commercial microporous polypropylene hollow fiber membranes	SEM for observing surface morphology and platelet depositions. XPS for surface chemistry. Colorimetric sulfo-SDTB amine assay for effectiveness of chitosan modification in increasing amine content. *In vitro* CO_2 removal test for evaluating application performance and the effect of chitosan coating on CO_2 permeance	[42]
Polyacrylonitrile-co-methyl methacrylate aminated by ethylene diamine at 99 °C	Urease/ Covalent attachment of lysine amine group of the enzyme on free aldehyde group on fiber surfaces.	Electrospun microfibrous mat with an average fiber diameter of 1448 ± 380 nm	SEM for morphology. FTIR for confirmation of chemical bonds. Activity assay for effect of crosslinker concentration, pH and temperature profiles, and kinetic parameters. Protein content in the filtrate was used to estimate enzyme loading.	[43]
Polymethyl pentene (PMP) aminated by polymerizing allylamine under plasma enhance chemical vapor deposition	Carbonic anhydrase/ surface amine groups were activated by glutaraldehyde followed by the attachment of a chitosan spacer layer. Amino groups on chitosan were in turn activated by glutaraldehyde and react with lysine amino groups on enzyme. All schiff's base linkages were reduced to secondary amine by sodium cyanoborohydride in each step.	Commercial poly (methyl pentene) (PMP) hollow fiber membranes (HFMs) (OD: 380 μm, ID: 200 μm)	CO_2 removal rate in a hollow fiber membrane reactor or oxygenator to evaluate the effect of dilute acid gas in oxygen sweep gas.	[44]
Poly (glycidyl methacrylate) (PGMA) grafted poly(vinyl alcohol) (PVA)	α-amylase / covalent attachment between amino groups on enzyme and epoxy group on PGMA brushes on the surface of the PVA nanofibers.	Photo cross-linked PVA nanofibers with diameter of 200-250 nm, after grafted with PGMA, diameter increased to ~550 nm.	SEM for morphology. NMR, FTIR, TGA, and XPS for confirming graft reaction. Activity assay for temperature and pH profile, reusability, and storage stability.	[45]

Polystyrene (PS) treated with nitric acid and silanized by 3-aminoproplytrimethoxysilane (APTMS)	Alcohol dehydrogenase/ amide linkages between free amino groups on the functionalized fiber surfaces and free carboxyl groups on the enzyme, or vice versa, both activated by EDC and NHS.	Electrospun PS with fiber diameters of 1.26±0.13 μm.	SEM for fiber morphology. EDX, FTIR, and Raman for confirming the functionalization reactions. Protein assay for enzyme loading. Activity assay for optimizing surface treatment recipe and storage stability. Fluorescence microscope for confirming enzyme attachment.	[46]
Poly (vinylidene fluoride) (PVDF) decorated with TiO2 and functionalized with 3-Amino-propyltriethoxysilane (APTES) and glutaraldehyde (GA)	Carbonic anhydrase/ adsorption or schiff's base linkage between amino groups on the support and enzymes by GA crosslinker.	Commercial Hydrophobic PVDF membranes with pore size of 0.45 μm.	SEM for morphology. Nitrogen adsorption–desorption measurements for surface area, pore diameter and pore volume of the membrane. TGA for TiO2 loading. Water contact angle and liquid entry pressure for membrane wettability. Activity assay for kinetic parameter, reusability, and storage stability. Streaming potential for isoelectric point (IEP) of the membrane.	[47]
Cellulose nanofiber (CNF) from kenaf	cyclodextrin glucanotranferase/ Covalent attachment between amine groups on enzyme and free aldehyde groups on glutaraldehyde activated dodecanediamine coated fiber surfaces.	CNF fiber diameter of less than 100 nm prepared by chemical-physical treatment including hot alkaline, bleaching and ultrasonication.	SEM and TEM for fiber morphology including diameter. FTIR for confirming chemical composition. HPLC-RI for quantifying reaction product, which is then used to calculate enzyme activity and production yield. Bradford protein assay for enzyme loading. Activity assay for temperature profile and reusability.	[48]
Collagen with Fe_3O_4 magnetic particle	Lipase/ Covalent attachment between collagen and enzyme using glutaraldehyde	Porous collagen/ Fe_3O_4 composite fibers with BET surface area of 11.59 m^2/g and pore size of 10.47 nm.	SEM for morphology. N_2 adsorption desorption for surface area, pore size, and pore volume. Activity assay for effects of immobilization condition, temperature and pH profiles, kinetic parameters, storage stability, and reusability. DSC and TGA for thermal stability.	[49]
Poly (ethylene terephthalate) (PET) hydrolyzed in acid to generate free carboxylic acid chain ends	Trypsin / Free carboxylic acid was activated by DCC and NHS forming activated ester which is then reacted with BSA acting as spacers. BSA was activated by glutaraldehyde whose free aldehyde is reacted to amine on Trypsin forming Schiff's base linkage.	100% PET plain weave woven fabric, yarn density of 92x70 yarn/inch, weight of 56.17 g/m^2, and thickness of 0.152 mm	Activity assay for optimization of recipe, storage stability, and reusability. SEM for fiber surface morphology. FTIR for confirming functionalization reactions.	[50]
Poly (lactic acid) (PLA) aminated using ammonia-based plasma treatment.	Trypsin/ Amine functionalized fiber surface was first activated by glutaraldehyde which is then reacted with amine groups on enzyme forming Schiff's base linkage.	100% PLA yarn: 84 dtex; Woven PLA: 75 denier, 56.83 g/m^2, 90x68 yarn/inch, 0.132 mm	X-ray photoelectron spectroscopy (XPS) for confirming surface chemistry change. SEM for fiber surface morphology. Protein assay for enzyme loading. Activity assay for optimizing immobilization recipe, temperature and pH	[51]

		thickness.	stability, storage stability, and reusability. Water contact angle and surface tension for surface hydrophilicity.	
Cotton treated with sodium periodate generating aldehyde groups on the surface	α-amylase and alkaline pectinase / amine groups on enzyme covalently bonded to aldehyde groups on cotton fibers	Pre-bleached knitted cotton fabric, 189 g/m^2	FTIR for surface chemistry change. DSC for thermal stability change due to periodate oxidation. Fehling test for detecting aldehyde. Antimicrobial activity test for application properties of the immobilized enzyme.	[52]
Poly (glycidyl methacrylate-co-methylacrylate)/ feather polypeptide	Lipase / covalent bonding between amine groups on enzyme and epoxy groups on the nanofiber membrane.	Electrospun nanofibrous membrane	FTIR for confirming the polymerization and immobilization reactions. Protein assay for enzyme loading. Activity assay for optimizing recipe, pH and temperature profiles, and for thermal stability, reusability, solvent stability, and kinetic parameters.	[53]
Cellulose	Glucose oxidase / amide bond formed between amine groups on enzyme and activated ester groups on one of the comonomers in the photocrosslinkable (benzophenone) copolymer coating on fiber surfaces.	Commercial filter paper: 84 g/m^2, BET surface area of ~1 m^2/g and mean pore diameter of 4.6 μm	FTIR for confirming polymerization and photocrosslinking reaction. Fluorescent microscopy for revealing the precise spatial control of the polymer coating and immobilization sites, and for visual comparison of enzyme activity.	[54]
Poly (vinyl alcohol) (PVA) reacted with 3-Chloropropinoylchloride and ethylenediamine in sequence creating amine functionalized surfaces	Alcohol dehydrogenase / Covalent coupling of amine on the fiber surfaces and amine on enzymes using glutaraldehyde.	Commercial PVA yarn: 250 dtex	FTIR for confirming the amination treatment produced desired functional groups. SEM for morphology. Activity assay for effect of spacer concentration and length, pH and thermal stability, and reusability.	[55]
Polyamide-6,6 (nylon-6,6) / chitosan blend	α-chymotrypsin or trypsin / carboxyl groups on enzyme were first activated by EDC/NHS to from activated ester which is reactive toward primary amine on fiber surfaces forming amide bond.	Electrospun nanofibers with fiber diameter of 130-143 nm. Pore size 31-36 nm.	SEM for morphologies before and after immobilization and fouling, Confocal layer scanning microscope (CLSM) for immobilized enzyme distribution. Activity assay for storage stability and reusability, membrane permeance properties for studying protein fouling.	[56]
Polyethylene/Polypropylene (PEPP) with plasma polymerized	Laccase / amine functionalized surfaced was activated with glutaraldehyde and then the free aldehyde reacts with amine groups on enzyme.	Nonwoven PEPP conjugated fiber made by needle-punching and thermal	XPS for surface chemistry changes, activity assay for thermal and pH stability, storage stability, reusability, and kinetic parameters. SEM for surface morphology. Dye decolorization test as application evaluation.	[57]

allylamine or cyclopropylamine on the surface.		bonding: 430 g/m^2, 4.0 mm thick, pore size 38 μm.		
Poly (vinylidene fluoride) (PVDF) coated by mixture of dopamine and polyethylenimine (PEI)	Carbonic anhydrase/ covalent crosslinks formed between enzymes and between and the amine functionalized membrane surfaces using gluta aldehyde.	Commercial PVDF hollow fiber membrane with outer diameter of 1.5×10^{-3} m and inner diameter of 0.8×10−3 m, water contact angle of 110^0, pore size of ~40nm, porosity of 85%.	SEM for morphology and pore size distribution. FTIR, EDX and XPS for surface chemistry change. Water contact angle for effect of coating on hydrophobicity. Activity assay for optimization of immobilization recipe and conditions, and storage stability. AFM for surface roughness. CO_2 absorption flux for application performance.	[58]
Polysulfone with functionalized multi-wall carbon nanotubes (MWCNT)	Laccase/ adsorption or covalent attachment through membrane surface aldehyde or activated ester groups.	Polysulfone membrane by co-spin-coating with MWCNT and sacrificial PVP.	FTIR for confirming functionalization reaction. Raman for interaction between immobilized enzyme and MWCNT. SEM for morphology. Activity assay for thermal stability and activity yield. Degradation of phenolic compounds to measure application performance.	[59]
Polyacrylonitrile (PAN) / Fe_3O_4	Peroxidase / Dopamine coating on the nanofibers generating free amine groups that can be activated by glutaraldehyde and covalently attached to amine groups on enzyme	Magnetic nanofiber membrane made by co-electrospun of PAN with Fe_3O_4 nanoparticles (NPs).	SEM for surface morphology, and TEM for nanoparticle distribution in nanofiber. FTIR for confirming functionalization reaction. TGA for thermal stability change and NP loading, magnetization hysteresis curves for application properties. Protein assay for enzyme loading. Activity assay for effect of NP loading and enzyme loading on relative activity, and pH profiles. Phenol removal efficiency as application performance and for testing reusability.	[60]
Polyethylene terephthalate (PET)	Peroxidase/ Covalent attachments of enzymes on Polyvinyl amine functionalized surfaces using glutaraldehyde	Commercial PET felt.	Inductively coupled plasma optical emission spectroscopy (ICP-OES) for enzyme loading from iron content. Gas chromatograph (GC) and mass spectroscopy (MS) for quantifying epoxidation product yield and reusability.	[61]
Cellulose acetate/ Cellulose triacetate	Bromelain/ covalent attachment by glutaraldehyde on aminosilanized fiber surfaces or entrapment in fiber matrix through co-electrospun from polymer solution.	Electrospun cellulose acetate/ cellulose triacetate 70%/30% blend with fiber diameter range of 93–206 nm	FTIR for confirming acetylation reaction. Titration for degree of substitution. DSC and TGA for studying the thermal properties of the polymer blend. Activity assay for activity yield and storage stability. Cell viability test,	[62]

			water/physiological fluids uptake and swelling test. Membrane weight loss for potential biomedical applications.	
Polylactic acid (PLA), or Polycaprolactone (PCL), or Polyhydroxybutyrate-co-hydroxyvalerate (PHBV)	Acetylcholinesterase/ physical adsorption on fiber surfaces or covalent crosslinking along with spacers like Bovine Serum Albumin (BSA) and Nafion using glutaraldehyde	Electrospun nano/ micro-fibrous membrane with fiber diameters: PCL 5.1 ± 0.4 µm, PHBV 2.8 ± 0.1 µm, PLA 551 ± 122 nm.	SEM for fiber diameter and morphology. Activity assay for effect of polymer types, substrate, and enzyme Concentration. Incubation time on the repeatability and detection limits of the pesticide sensor application.	[63]
**Covalent attachment then physical entrapment**				
Poly (acrylic acid-co-acrylamide)/hydrotalcite (PAA-AAm/HT) nanocomposite hydrogels filling the interstitial space between PVDF hollow fibers	Bovine carbonic anhydrase type II/Both covalently bonded to PAA units through Carbodiimide-NHS activation and entrapped in the pores of the hydrogel.	Hydrophobic microporous PVDF hollow fiber with 60% porosity, 220 nm×380 nm pores, and a tortuosity value of 1.5	TEM for exfoliated HT layers, cryo-SEM for hydrogel porous structure, and SEM for hollow fiber member structure. Fluorescence microscopy for enzyme distribution. Hydrase activity by pH change for assay scale activity and solvent and thermal stability. Hollow fiber membrane reactor for application tests.	[64,65]
**Physical entrapment/ incorporation**				
Sodium alginate + Various divalent metallic ions	Glucoamylase; CGTase; Endo-Polygalacturonase; Protease; Glucose oxidase; Peroxidase/entrapped in alginate fiber	Wet-spun fibers, which were cut and processed into papers	Entrapment yield. Paper breaking length.	[66]
Silk fibroin; Viscose rayon; Polyethyleneterephthalate; Nylon-6; Polypropylene (fluorine treated)	Glucose Oxidase/ entrapped in silk fibroin coating	Silk fibroin coated on various nonwoven fabrics	Weight Increase. Apparent Michaelis Constant. Apparent Maximum Activities. Activity Yield. Spin labeling ESR for estimating amount of immobilized enzyme.	[67]
PET and Nylon 6,6	Catalase/Entrapped in UV-cured resin (1,4-Cyclohexanedimethanol divinyl ether or Diallyl phthalate) on fiber surfaces.	Commercial textile fabrics	SEM for morphology. Activity assay for activity retention compared to the free enzyme and after repeated use. FTIR, UV-Vis, and Ninhydrin test to qualitatively prove	[68]

			enzyme immobilization. Atomic absorption spectroscopy to quantify enzyme loading.	
Poly (ethylene oxide) (PEO) or Poly(ε-caprolactone) (PCL)	diisopropylfluorophosphatase (DFPase)/entrapped in PEO matrix or forming sheath on the PEO or PCL fiber core.	Core-shell electrospun nanofiber mat with fiber diameter of 120-218 nm.	TEM for core-sheath structure. SEM for fiber morphology. EDX for detecting enzymes containing sulfur.	[69]
Poly (vinyl alcohol) (PVA)	Lipase/Entrapped in PVA fibers along with substrate-mimicking additives for bio-imprinting.	Electrospun nanofibers with an average diameter of 500 nm.	SEM for morphology. Gas chromatography for catalytic reaction product analysis, activity, and reusability test. Zero-shear viscosity measurement of spinning dope. DSC for effect of additive on Tg of PVA.	[70]
poly(ε-caprolactone) (PCL) w/ or w/o γ-CD	Laccase/ Entrapment in PCL fibers w/ or w/o γ-CD by co-electrospinning the mixture.	Electrospun PCL nanofiber mesh	XRD for detecting possible inclusion complex formation. FTIR for chemical composition. SEM for morphology. Activity assay for comparing various immobilization technique variations.	[71]
**Physical entrapment then crosslinking**				
Poly (vinyl alcohol)	Lipase/entrapment in PVA nanofibers, crosslinked in GA ethanol solution at low pH.	Electrospun nanofibrous membrane with fiber diameter of 500 nm.	SEM for morphology and fiber diameter. Solution viscosity of spinning dope. DSC and TGA for interaction of lipase and PVA in the solid state. Swelling ratio for degree of crosslinking. Activity assay for thermal and storage stability, effect of ethanol and GA crosslinking.	[72]
Polyvinyl alcohol (PVA)/Bovine serum albumin (BSA)	Horseradish peroxidase/ Entrapped in PVA/BSA composite fiber matrix and cross-linked by glutaraldehyde vapor through formation of acetal and Schiff's base linkages.	Electrospun fiber with diameter of ~ 300 nm.	SEM for fiber morphology. Bradford protein assay for BAS release kinetics. Activity assay for formulation optimization, reusability, and kinetic parameters.	[73]
**Simultaneous incorporation and crosslinking**				
Polyester with porous crosslinked chitosan coating	Horseradish peroxidase/ adsorption on surface of and in the pores of chitosan matrices and possibly covalently bonded to the free aldehyde from GA cross-linker used in the coating process.	Nonwoven polyester fabric	Activity assay for temperature and pH profiles, thermal and pH stabilities, reusability, and effects of denaturants, surfactant, and organic solvents.	[74]

PVA crosslinked with glutaraldehyde	α-galactosidase/ two-step method: entrapment in the PVA nanofiber and subsequently crosslinked by glutaraldehyde in acetone. Or single-step in-situ crosslinking of PVA and enzyme during electrospinning without using a non-solvent.	Electrospun nanofibers with average diameter of ~280 nm.	SEM for fiber morphology. Activity assay for thermal stability, effects of solvent and buffer, pH, crosslinker, cryoprotectant, enzyme loading and mat thickness on apparent activity.	[75,76]
**Sacrificial matrix**				
PEO as temporary support	Cellulase/pure enzyme core cross-linked by glutaraldehyde vapor before temporary PEO sheath was washed away.	Cross-linked enzyme nanofibers with fiber diameter of 100-500 nm.	SEM for morphology and EDX for enzyme distribution through complexed Ca^{2+}. FTIR for detecting polymer and enzyme. Activity assay for kinetic parameters.	[77]

References

1. Demirkan, E.; Avci, T.; Aykut, Y. Protease Immobilization on Cellulose Monoacetate/Chitosan-Blended Nanofibers. *J. Ind. Text.* **2018**, *47*, 2092–2111, doi:10.1177/1528083717720205.
2. Magne, V.; Amounas, M.; Innocent, C.; Dejean, E.; Seta, P. Enzyme Textile for Removal of Urea with Coupling Process: Enzymatic Reaction and Electrodialysis. *Desalination* **2002**, *144*, 163–166.
3. Chen, X.; Wang, Y.; Wang, P. Peptide-Induced Affinity Binding of Carbonic Anhydrase to Carbon Nanotubes. *Langmuir* **2015**, *31*, 397–403, doi:10.1021/la504321q.
4. Debeche, T.; Marmet, C.; Kiwi-Minsker, L.; Renken, A.; Juillerat, M.-A. Structured Fiber Supports for Gas Phase Biocatalysis. *Enzyme Microb. Technol.* **2005**, *36*, 911–916, doi:10.1016/j.enzmictec.2005.01.012.
5. Wang, Z.-G.; Wang, J.-Q.; Xu, Z.-K. Immobilization of Lipase from Candida Rugosa on Electrospun Polysulfone Nanofibrous Membranes by Adsorption. *J. Mol. Catal. B Enzym.* **2006**, *42*, 45–51, doi:10.1016/j.molcatb.2006.06.004.
6. Ghosh, S.; Chaganti, S.R.; Prakasham, R.S. Polyaniline Nanofiber as a Novel Immobilization Matrix for the Anti-Leukemia Enzyme l-Asparaginase. *J. Mol. Catal. B Enzym.* **2012**, *74*, 132–137, doi:10.1016/j.molcatb.2011.09.009.
7. Tavares, A.P.M.; Silva, C.G.; Dražić, G.; Silva, A.M.T.; Loureiro, J.M.; Faria, J.L. Laccase Immobilization over Multi-Walled

Carbon Nanotubes: Kinetic, Thermodynamic and Stability Studies. *J. Colloid Interface Sci.* **2015**, *454*, 52–60, doi:10.1016/j.jcis.2015.04.054.

8. Zhang, J.; Song, M.; Wang, X.; Wu, J.; Yang, Z.; Cao, J.; Chen, Y.; Wei, Q. Preparation of a Cellulose Acetate/Organic Montmorillonite Composite Porous Ultrafine Fiber Membrane for Enzyme Immobilization. *J. Appl. Polym. Sci.* **2016**, *133*, doi:10.1002/app.43818.
9. Yoshimoto, M.; Schweizer, T.; Rathlef, M.; Pleij, T.; Walde, P. Immobilization of Carbonic Anhydrase in Glass Micropipettes and Glass Fiber Filters for Flow-Through Reactor Applications. *ACS Omega* **2018**, *3*, 10391–10405, doi:10.1021/acsomega.8b01517.
10. Solas, M.T.; Vicente, C.; Xavier, L.; Legaz, M.E. Ionic Adsorption of Catalase on Bioskin: Kinetic and Ultrastructural Studies. *J. Biotechnol.* **1994**, *33*, 63–70, doi:10.1016/0168-1656(94)90099-X.
11. Karimpil, J.J.; Melo, J.S.; D'Souza, S.F. Immobilization of Lipase on Cotton Cloth Using the Layer-by-Layer Self-Assembly Technique. *Int. J. Biol. Macromol.* **2012**, *50*, 300–302, doi:10.1016/j.ijbiomac.2011.10.019.
12. Liu, J.; Wang, Q.; Fan, X.R.; Sun, X.J.; Huang, P.H. Layer-by-Layer Self-Assembly Immobilization of Catalases on Wool Fabrics. *Appl. Biochem. Biotechnol.* **2013**, *169*, 2212–2222, doi:10.1007/s12010-013-0093-6.
13. Yong, J.K.J.; Cui, J.; Cho, K.L.; Stevens, G.W.; Caruso, F.; Kentish, S.E. Surface Engineering of Polypropylene Membranes with Carbonic Anhydrase-Loaded Mesoporous Silica Nanoparticles for Improved Carbon Dioxide Hydration. *Langmuir* **2015**, *31*, 6211–6219.
14. Mohamad, N.; Buang, N.A.; Mahat, N.A.; Jamalis, J.; Huyop, F.; Aboul-Enein, H.Y.; Wahab, R.A. Simple Adsorption of Candida Rugosa Lipase onto Multi-Walled Carbon Nanotubes for Sustainable Production of the Flavor Ester Geranyl Propionate. *J. Ind. Eng. Chem.* **2015**, *32*, 99–108, doi:10.1016/j.jiec.2015.08.001.
15. Fu, J.; Li, D.; Li, G.; Huang, F.; Wei, Q. Carboxymethyl Cellulose Assisted Immobilization of Silver Nanoparticles onto Cellulose Nanofibers for the Detection of Catechol. *J. Electroanal. Chem.* **2015**, *738*, 92–99, doi:10.1016/j.jelechem.2014.11.025.
16. Yong, J.K.J.; Stevens, G.W.; Caruso, F.; Kentish, S.E. In Situ Layer-by-Layer Assembled Carbonic Anhydrase-Coated Hollow Fiber Membrane Contactor for Rapid CO2 Absorption. *J. Memb. Sci.* **2016**, *514*, 556–565, doi:10.1016/j.memsci.2016.05.020.
17. Wu, R.; Zhou, D.; Wang, G. Preparation of Immobilized Enzymes on Pulp Fiber with the Layer-by-Layer Self-Assembly Technique and Its Application in Whitewater Treatment. *BioResources* **2018**, *13*, 8455–8463, doi:10.15376/biores.13.4.8455-8463.
18. Kim, H.; Lee, I.; Kwon, Y.; Kim, B.C.; Ha, S.; Lee, J. heon; Kim, J. Immobilization of Glucose Oxidase into Polyaniline Nanofiber Matrix for Biofuel Cell Applications. *Biosens. Bioelectron.* **2011**, *26*, 3908–3913, doi:10.1016/j.bios.2011.03.008.
19. Sahoo, P.C.; Sambudi, N.S.; Park, S. Bin; Lee, J.H.; Han, J.-I. Immobilization of Carbonic Anhydrase on Modified Electrospun Poly(Lactic Acid) Membranes: Quest for Optimum Biocatalytic Performance. *Catal. Letters* **2015**, *145*, 519–526, doi:10.1007/s10562-014-1406-2.

20. İspirli Doğaç, Y.; Deveci, İ.; Mercimek, B.; Teke, M. A Comparative Study for Lipase Immobilization onto Alginate Based Composite Electrospun Nanofibers with Effective and Enhanced Stability. *Int. J. Biol. Macromol.* **2017**, *96*, 302–311, doi:10.1016/j.ijbiomac.2016.11.120.
21. Pialis, P.; Jimenez Hamann, M.C.; Seville, B.A. L-DOPA Production from Tyrosinase Immobilized on Nylon 6,6. *Biotechnol. Bioeng.* **1996**, *51*, 141–147, doi:10.1002/(SICI)1097-0290(19960720)51:2<141::AID-BIT2>3.0.CO;2-J.
22. Pialis, P.; Saville, B.A. Production of L-DOPA from Tyrosinase Immobilized on Nylon 6,6: Enzyme Stability and Scaleup. *Enzyme Microb. Technol.* **1998**, *22*, 261–268, doi:10.1016/S0141-0229(97)00195-6.
23. Edwards, J.V.; Sethumadhavan, K.; Ullah, A.H.J. Conjugation and Modeled Structure/Function Analysis of Lysozyme on Glycine Esterified Cotton Cellulose-Fibers. *Bioconjug. Chem.* **2000**, *11*, 469–473, doi:10.1021/bc990141u.
24. Jia, H.; Zhu, G.; Vugrinovich, B.; Kataphinan, W.; Reneker, D.H.; Wang, P. Enzyme-carrying Polymeric Nanofibers Prepared via Electrospinning for Use as Unique Biocatalysts. *Biotechnol. Prog.* **2002**, *18*, 1027–1032.
25. Opwis, K.; Knittel, D.; Schollmeyer, E. Quantitative Analysis of Immobilized Metalloenzymes by Atomic Absorption Spectroscopy. *Anal. Bioanal. Chem.* **2004**, *380*, 937–941, doi:10.1007/s00216-004-2875-8.
26. Wang, Y.; Hsieh, Y. Enzyme Immobilization to Ultra-Fine Cellulose Fibers via Amphiphilic Polyethylene Glycol Spacers. *J. Polym. Sci. Part A Polym. Chem.* **2004**, *42*, 4289–4299, doi:10.1002/pola.20271.
27. Kim, B.C.; Nair, S.; Kim, J.; Kwak, J.H.; Grate, J.W.; Kim, S.H.; Gu, M.B. Preparation of Biocatalytic Nanofibres with High Activity and Stability via Enzyme Aggregate Coating on Polymer Nanofibres. *Nanotechnology* **2005**, *16*, doi:10.1088/0957-4484/16/7/011.
28. Yeon, K.-H.; Lueptow, R.M. Urease Immobilization on an Ion-Exchange Textile for Urea Hydrolysis. *J. Chem. Technol. Biotechnol.* **2006**, *81*, 940–950, doi:10.1002/jctb.1471.
29. Ye, P.; Xu, Z.K.; Wu, J.; Innocent, C.; Seta, P. Nanofibrous Poly(Acrylonitrile-Co-Maleic Acid) Membranes Functionalized with Gelatin and Chitosan for Lipase Immobilization. *Biomaterials* **2006**, *27*, 4169–4176, doi:10.1016/j.biomaterials.2006.03.027.
30. Silva, C.; Silva, C.J.; Zille, A.; Guebitz, G.M.; Cavaco-Paulo, A. Laccase Immobilization on Enzymatically Functionalized Polyamide 6,6 Fibres. *Enzyme Microb. Technol.* **2007**, *41*, 867–875, doi:10.1016/j.enzmictec.2007.07.010.
31. Ibrahim, N.A.; Gouda, M.; El-shafei, A.M.; Abdel-Fatah, O.M. Antimicrobial Activity of Cotton Fabrics Containing Immobilized Enzymes. *J. Appl. Polym. Sci.* **2007**, *104*, 1754–1761, doi:10.1002/app.25821.
32. Nair, S.; Kim, J.; Crawford, B.; Kim, S.H. Improving Biocatalytic Activity of Enzyme-Loaded Nanofibers by Dispersing Entangled Nanofiber Structure. *Biomacromolecules* **2007**, *8*, 1266–1270, doi:10.1021/bm061004k.
33. Li, S.-F.; Chen, J.-P.; Wu, W.-T. Electrospun Polyacrylonitrile Nanofibrous Membranes for Lipase Immobilization. *J. Mol. Catal. B Enzym.* **2007**, *47*, 117–124.
34. Wang, Q.; Li, C.X.; Fan, X.; Wang, P.; Cui, L. Immobilization of Catalase on Cotton Fabric Oxidized by Sodium Periodate.

Biocatal. Biotransformation **2008**, *26*, 437–443, doi:10.1080/10242420802335031.
35. Wang, Q.; Fan, X.; Hu, Y.; Yuan, J.; Cui, L.; Wang, P. Antibacterial Functionalization of Wool Fabric via Immobilizing Lysozymes. *Bioprocess Biosyst. Eng.* **2009**, *32*, 633–639, doi:10.1007/s00449-008-0286-5.
36. Edwards, J.V.; Prevost, N.T.; Condon, B.; French, A. Covalent Attachment of Lysozyme to Cotton/Cellulose Materials: Protein Verses Solid Support Activation. *Cellulose* **2011**, *18*, 1239–1249, doi:10.1007/s10570-011-9563-6.
37. Dai, T.; Miletić, N.; Loos, K.; Elbahri, M.; Abetz, V. Electrospinning of Poly [Acrylonitrile-co-(Glycidyl Methacrylate)] Nanofibrous Mats for the Immobilization of Candida Antarctica Lipase B. *Macromol. Chem. Phys.* **2011**, *212*, 319–327.
38. Arazawa, D.T.; Oh, H. Il; Ye, S.H.; Johnson, C.A.; Woolley, J.R.; Wagner, W.R.; Federspiel, W.J. Immobilized Carbonic Anhydrase on Hollow Fiber Membranes Accelerates CO2 Removal from Blood. *J. Memb. Sci.* **2012**, *403–404*, 25–31, doi:10.1016/j.memsci.2012.02.006.
39. Arola, S.; Tammelin, T.; Setälä, H.; Tullila, A.; Linder, M.B. Immobilization–Stabilization of Proteins on Nanofibrillated Cellulose Derivatives and Their Bioactive Film Formation. *Biomacromolecules* **2012**, *13*, 594–603.
40. Zhu, J.; Sun, G. Lipase Immobilization on Glutaraldehyde-Activated Nanofibrous Membranes for Improved Enzyme Stabilities and Activities. *React. Funct. Polym.* **2012**, *72*, 839–845, doi:10.1016/j.reactfunctpolym.2012.08.001.
41. Park, J.-M.; Kim, M.; Park, H.-S.; Jang, A.; Min, J.; Kim, Y.-H. Immobilization of Lysozyme-CLEA onto Electrospun Chitosan Nanofiber for Effective Antibacterial Applications. *Int. J. Biol. Macromol.* **2013**, *54*, 37–43, doi:10.1016/j.ijbiomac.2012.11.025.
42. Kimmel, J.D.; Arazawa, D.T.; Ye, S.H.; Shankarraman, V.; Wagner, W.R.; Federspiel, W.J. Carbonic Anhydrase Immobilized on Hollow Fiber Membranes Using Glutaraldehyde Activated Chitosan for Artificial Lung Applications. *J. Mater. Sci. Mater. Med.* **2013**, *24*, 2611–2621, doi:10.1007/s10856-013-5006-2.
43. Daneshfar, A.; Matsuura, T.; Emadzadeh, D.; Pahlevani, Z.; Ismail, A.F. Urease-Carrying Electrospun Polyacrylonitrile Mat for Urea Hydrolysis. *React. Funct. Polym.* **2015**, *87*, 37–45, doi:10.1016/j.reactfunctpolym.2014.12.004.
44. Arazawa, D.T.; Kimmel, J.D.; Finn, M.C.; Federspiel, W.J. Acidic Sweep Gas with Carbonic Anhydrase Coated Hollow Fiber Membranes Synergistically Accelerates CO2 Removal from Blood. *Acta Biomater.* **2015**, *25*, 143–149, doi:10.1016/j.actbio.2015.07.007.
45. Oktay, B.; Demir, S.; Kayaman-Apohan, N. Immobilization of α-Amylase onto Poly(Glycidyl Methacrylate) Grafted Electrospun Fibers by ATRP. *Mater. Sci. Eng. C* **2015**, *50*, 386–393, doi:10.1016/j.msec.2015.02.033.
46. An, H.; Jin, B.; Dai, S. Fabricating Polystyrene Fiber-Dehydrogenase Assemble as a Functional Biocatalyst. *Enzyme Microb. Technol.* **2015**, *68*, 15–22.
47. Hou, J.; Dong, G.; Xiao, B.; Malassigne, C.; Chen, V. Preparation of Titania Based Biocatalytic Nanoparticles and Membranes for CO2 Conversion. *J. Mater. Chem. A* **2015**, *3*, 3332–3342, doi:10.1039/C4TA05760K.
48. Sulaiman, S.; Cieh, N.L.; Mokhtar, M.N.; Naim, M.N.; Kamal, S.M.M. Covalent Immobilization of Cyclodextrin

Glucanotransferase on Kenaf Cellulose Nanofiber and Its Application in Ultrafiltration Membrane System. *Process Biochem.* **2017**, *55*, 85–95, doi:10.1016/j.procbio.2017.01.025.
49. He, S.; Song, D.; Chen, M.; Cheng, H. Immobilization of Lipases on Magnetic Collagen Fibers and Its Applications for Short-Chain Ester Synthesis. *Catalysts* **2017**, *7*, 178, doi:10.3390/catal7060178.
50. Shim, E.J.; Lee, S.H.; Song, W.S.; Kim, H.R. Development of an Enzyme-Immobilized Support Using a Polyester Woven Fabric. *Text. Res. J.* **2017**, *87*, 3–14, doi:10.1177/0040517515624874.
51. Song, J.E.; Song, W.S.; Yeo, S.Y.; Kim, H.R.; Lee, S.H. Covalent Immobilization of Enzyme on Aminated Woven Poly (Lactic Acid) via Ammonia Plasma: Evaluation of the Optimum Immobilization Conditions. *Text. Res. J.* **2017**, *87*, 1177–1191, doi:10.1177/0040517516648514.
52. Coradi, M.; Zanetti, M.; Valério, A.; de Oliveira, D.; da Silva, A.; Maria de Arruda Guelli Ulson de Souza, S.; Ulson de Souza, A.A. Production of Antimicrobial Textiles by Cotton Fabric Functionalization and Pectinolytic Enzyme Immobilization. *Mater. Chem. Phys.* **2018**, *208*, 28–34, doi:10.1016/j.matchemphys.2018.01.019.
53. Liu, X.; Fang, Y.; Yang, X.; Li, Y.; Wang, C. Electrospun Epoxy-Based Nanofibrous Membrane Containing Biocompatible Feather Polypeptide for Highly Stable and Active Covalent Immobilization of Lipase. *Colloids Surfaces B Biointerfaces* **2018**, *166*, 277–285.
54. Böhm, A.; Trosien, S.; Avrutina, O.; Kolmar, H.; Biesalski, M. Covalent Attachment of Enzymes to Paper Fibers for Paper-Based Analytical Devices. *Front. Chem.* **2018**, *6*.
55. Shinde, P.; Musameh, M.; Gao, Y.; Robinson, A.J.; Kyratzis, I. (Louis) Immobilization and Stabilization of Alcohol Dehydrogenase on Polyvinyl Alcohol Fibre. *Biotechnol. Reports* **2018**, *19*, e00260, doi:10.1016/j.btre.2018.e00260.
56. Vanangamudi, A.; Saeki, D.; Dumée, L.F.; Duke, M.; Vasiljevic, T.; Matsuyama, H.; Yang, X. Surface-Engineered Biocatalytic Composite Membranes for Reduced Protein Fouling and Self-Cleaning. *ACS Appl. Mater. Interfaces* **2018**, *10*, 27477–27487.
57. Lee, S.H.; Yeo, S.Y.; Cools, P.; Morent, R. Plasma Polymerization onto Nonwoven Polyethylene/Polypropylene Fibers for Laccase Immobilization as Dye Decolorization Filter Media. *Text. Res. J.* **2019**, *89*, 3578–3590, doi:10.1177/0040517518817102.
58. Xu, Y.; Lin, Y.; Chew, N.G.P.; Malde, C.; Wang, R. Biocatalytic PVDF Composite Hollow Fiber Membranes for CO2 Removal in Gas-Liquid Membrane Contactor. *J. Memb. Sci.* **2019**, *572*, 532–544, doi:10.1016/j.memsci.2018.11.043.
59. Costa, J.B.; Lima, M.J.; Sampaio, M.J.; Neves, M.C.; Faria, J.L.; Morales-Torres, S.; Tavares, A.P.M.; Silva, C.G. Enhanced Biocatalytic Sustainability of Laccase by Immobilization on Functionalized Carbon Nanotubes/Polysulfone Membranes. *Chem. Eng. J.* **2019**, *355*, 974–985.
60. Li, J.; Chen, X.; Xu, D.; Pan, K. Immobilization of Horseradish Peroxidase on Electrospun Magnetic Nanofibers for Phenol Removal. *Ecotoxicol. Environ. Saf.* **2019**, *170*, 716–721.
61. Wunschik, D.S.; Ingenbosch, K.N.; Süss, P.; Liebelt, U.; Quint, S.; Dyllick-Brenzinger, M.; Zuhse, R.; Menyes, U.; Hoffmann-

Jacobsen, K.; Opwis, K.; et al. Enzymatic Epoxidation of Cyclohexene by Peroxidase Immobilization on a Textile and an Adapted Reactor Design. *Enzyme Microb. Technol.* **2020**, *136*, 109512, doi:10.1016/j.enzmictec.2020.109512.
62. de Melo Brites, M.; Cerón, A.A.; Costa, S.M.; Oliveira, R.C.; Ferraz, H.G.; Catalani, L.H.; Costa, S.A. Bromelain Immobilization in Cellulose Triacetate Nanofiber Membranes from Sugarcane Bagasse by Electrospinning Technique. *Enzyme Microb. Technol.* **2020**, *132*, 109384, doi:10.1016/j.enzmictec.2019.109384.
63. Cacciotti, I.; Pallotto, F.; Scognamiglio, V.; Moscone, D.; Arduini, F. Reusable Optical Multi-Plate Sensing System for Pesticide Detection by Using Electrospun Membranes as Smart Support for Acetylcholinesterase Immobilisation. *Mater. Sci. Eng. C* **2020**, *111*, 110744, doi:10.1016/j.msec.2020.110744.
64. Zhang, Y.-T.; Zhang, L.; Chen, H.-L.; Zhang, H.-M. Selective Separation of Low Concentration CO2 Using Hydrogel Immobilized CA Enzyme Based Hollow Fiber Membrane Reactors. *Chem. Eng. Sci.* **2010**, *65*, 3199–3207, doi:10.1016/j.ces.2010.02.010.
65. Zhang, Y.T.; Zhi, T.T.; Zhang, L.; Huang, H.; Chen, H.L. Immobilization of Carbonic Anhydrase by Embedding and Covalent Coupling into Nanocomposite Hydrogel Containing Hydrotalcite. *Polymer (Guildf).* **2009**, *50*, 5693–5700, doi:10.1016/j.polymer.2009.09.067.
66. Kobayashi, Y.; Matsuo, R.; Ohya, T.; Yokoi, N. Enzyme-Entrapping Behaviors in Alginate Fibers and Their Papers. *Biotechnol. Bioeng.* **1987**, *30*, 451–457, doi:10.1002/bit.260300316.
67. Asakura, T.; Kitaguchi, M.; Demura, M.; Sakai, H.; Komatsu, K. Immobilization of Glucose Oxidase on Nonwoven Fabrics with Bombyx Mori Silk Fibroin Gel. *J. Appl. Polym. Sci.* **1992**, *46*, 49–53, doi:10.1002/app.1992.070460106.
68. Opwis, K.; Knittel, D.; Bahners, T.; Schollmeyer, E. Photochemical Enzyme Immobilization on Textile Carrier Materials. *Eng. Life Sci.* **2005**, *5*, 63–67, doi:10.1002/elsc.200420058.
69. Han, D.; Filocamo, S.; Kirby, R.; Steckl, A.J. Deactivating Chemical Agents Using Enzyme-Coated Nanofibers Formed by Electrospinning. *ACS Appl. Mater. Interfaces* **2011**, *3*, 4633–4639, doi:10.1021/am201064b.
70. Weiser, D.; Sóti, P.L.; Bánóczi, G.; Bódai, V.; Kiss, B.; Gellért, Á.; Nagy, Z.K.; Koczka, B.; Szilágyi, A.; Marosi, G.; et al. Bioimprinted Lipases in PVA Nanofibers as Efficient Immobilized Biocatalysts. *Tetrahedron* **2016**, *72*, 7335–7342, doi:10.1016/j.tet.2016.06.027.
71. Canbolat, M.F.; Savas, H.B.; Gultekin, F. Enzymatic Behavior of Laccase Following Interaction with γ-CD and Immobilization into PCL Nanofibers. *Anal. Biochem.* **2017**, *528*, 13–18, doi:10.1016/j.ab.2017.04.005.
72. Wang, Y.; Hsieh, Y.-L. Immobilization of Lipase Enzyme in Polyvinyl Alcohol (PVA) Nanofibrous Membranes. *J. Memb. Sci.* **2008**, *309*, 73–81, doi:10.1016/j.memsci.2007.10.008.
73. Fazel, R.; Torabi, S.-F.; Naseri-Nosar, P.; Ghasempur, S.; Ranaei-Siadat, S.-O.; Khajeh, K. Electrospun Polyvinyl Alcohol/Bovine Serum Albumin Biocomposite Membranes for Horseradish Peroxidase Immobilization. *Enzyme Microb. Technol.* **2016**, *93–94*, 1–

10, doi:10.1016/j.enzmictec.2016.07.002.
74. Mohamed, S.A.; Aly, A.S.; Mohamed, T.M.; Salah, H.A. Immobilization of Horseradish Peroxidase on Nonwoven Polyester Fabric Coated with Chitosan. *Appl. Biochem. Biotechnol.* **2008**, *144*, 169–179, doi:10.1007/s12010-007-8026-x.
75. Tang, C.; Saquing, C.D.; Morton, S.W.; Glatz, B.N.; Kelly, R.M.; Khan, S.A. Cross-Linked Polymer Nanofibers for Hyperthermophilic Enzyme Immobilization: Approaches to Improve Enzyme Performance. *ACS Appl. Mater. Interfaces* **2014**, *6*, 11899–11906, doi:10.1021/am5033633.
76. Tang, C.; Saquing, C.D.; Sarin, P.K.; Kelly, R.M.; Khan, S.A. Nanofibrous Membranes for Single-Step Immobilization of Hyperthermophilic Enzymes. *J. Memb. Sci.* **2014**, *472*, 251–260, doi:10.1016/j.memsci.2014.08.037.
77. Tran, D.N.; Yang, D.J.; Balkus, K.J. Fabrication of Cellulase Protein Fibers through Concentric Electrospinning. *J. Mol. Catal. B Enzym.* **2011**, *72*, 1–5, doi:10.1016/j.molcatb.2011.04.001.
